# Attitudes of healthcare professionals towards persons with disabilities: a systematic review (2015–2025)

**DOI:** 10.1186/s12909-026-09922-0

**Published:** 2026-07-15

**Authors:** Iwona Radlińska, Gustaw Radliński, Arkadiusz Prajzner, Karolina Dymek, Wiktoria Soprych, Łukasz Krzywoszański

**Affiliations:** 1 Department of Social Medicine, Pomeranian Medical University in Szczecin, Subdepartment of Medical Law, Ul. Żołnierska 48, 71-210 Szczecin, Poland; 2 Student’s Science Club of Data Analysis, Krakow University of Economics, Ul. Rakowicka 27, 31-510 Kraków, Poland; 3 Faculty of Pedagogy and Psychology, University of the National Education Commission, Institute of Psychology, Ul. Podchorążych 2, 30-084 Kraków, Poland; 4 Doctoral School, University of the National Education Commission, Kraków, Poland

**Keywords:** Ableism, Attitudes, Disability, Health care professionals, Inclusive healthcare, Paternalism

## Abstract

**Background:**

The attitudes of healthcare professionals towards persons with disabilities are a key determinant of the quality, accessibility, and equality of healthcare. Despite increasing recognition of ableism in healthcare, systematic reviews that synthesise these attitudes across medical professions remain scarce. This systematic review aimed to examine healthcare professionals’ attitudes towards persons with various types of disability, including intellectual disability, physical disability, and disability in general, defined as disability without specification of type, and to identify factors associated with these attitudes.

**Methods:**

Four electronic databases (PubMed, Web of Science, ERIC, and ProQuest) were systematically searched for English-language studies published from January 2015 to February 2025. Supplementary backward and forward citation searching continued until May 2025. After duplicate removal, 1,690 records were screened. Study selection was completed in May 2025, resulting in the inclusion of 27 studies, most of which were cross-sectional and of good overall quality. Quantitative and mixed-methods studies were included. Data were synthesised narratively, taking into account professional groups, countries, clinical contexts, and final disability categories: intellectual disability, physical/sensory disabilities, and disability in general. Reporting quality and risk of bias were assessed using the STROBE-M checklist.

**Results:**

Across the 27 included studies, overall attitudes were moderately positive but varied substantially. The greatest challenges were observed in relation to intellectual disabilities, where ambivalence, paternalistic attitudes, and the undermining of patients’ competence were more frequently reported. The key predictors of positive attitudes included previous professional experience, the quality of contact with persons with disabilities, and formal training in disability-related issues. Marked differences were identified between countries and professional groups, highlighting the role of cultural context and systemic preparation.

**Conclusions:**

The findings underscore the need for contact-based educational interventions, the development of clear policy standards to counter ableism, and further research into implicit bias in healthcare settings. Implementing these measures may enhance the quality and inclusiveness of care provided to persons with disabilities. The evidence is limited by the predominance of self-reported measures and the use of author-developed questionnaires in some studies. Funding: No external funding. Registration: Not registered.

**Supplementary Information:**

The online version contains supplementary material available at https://doi.org/10.1186/s12909-026-09922-0.

## Background

### Social exclusion as a context for attitudes towards persons with disabilities

The World Health Organization (WHO) estimates that approximately 1.3 billion people, or around 16% of the world’s population, live with significant disabilities [1]. In response to persistent health inequities, WHO promotes health equity through disability inclusion within the health sector [2]. However, the 2022 WHO Global Report on health equity for persons with disabilities indicates that the world remains far from realising the right to the highest attainable standard of health for many persons with disabilities [1]. These individuals experience poorer health outcomes than the general population, including earlier mortality, poorer overall health, and greater limitations in daily functioning, due to structural and systemic inequalities affecting all areas of life, including healthcare systems [1]. This is in line with the United Nations Convention on the Rights of Persons with Disabilities (UNCRPD)**,** adopted on 6 December 2006 [3], which emphasises the need to raise awareness of the rights of persons with disabilities and to combat stereotypes, prejudices, and harmful practices. Research from the last decade has consistently demonstrated that persons with disabilities (PwD) frequently experience social exclusion, isolation, and loneliness. Evidence from systematic reviews and meta-analyses indicates that individuals with disabilities report significantly higher levels of loneliness and lower overall well-being compared to those without disabilities [4–6], with social barriers and a lack of inclusive structures considered key contributing factors [5]. This broader context is relevant to the present review because social and institutional attitudes, including those held by healthcare professionals, may contribute to barriers in access to care, patient–provider communication, and the quality of healthcare received by persons with disabilities.

In this review, attitudes are understood as multidimensional evaluations that include cognitive components (knowledge, beliefs, and stereotypes), affective components (emotions and feelings), and behavioural components (intentions, actions, and patterns of interaction) [7, 8]. Attitudes towards disabled people may be positive, neutral or negative. Positive attitudes may involve respect, recognition of autonomy, empathy and support for inclusion. Negative attitudes, on the other hand, may include unfavourable beliefs, discomfort, fear, stereotypes, avoidance and lower expectations regarding the abilities and autonomy of persons with disabilities [9, 10]. In healthcare settings, such attitudes may impact patient-provider communication, clinical decision-making, and the extent to which persons with disabilities are treated as autonomous participants in care [9, 10].

In this review, “stigmatising attitudes” are understood more specifically as negative attitudes embedded in a broader stigma process involving labelling, stereotyping, separation, status loss, and discrimination [11]. Thus, not every negative attitude necessarily constitutes stigma; however, negative attitudes may become stigmatising when they reinforce social distance, unequal treatment, or restrictions on autonomy [9, 11].

### Negative attitudes as a factor contributing to marginalisation

It is worth emphasising that the challenges faced by persons with disabilities (PwD), such as social exclusion, isolation, and loneliness, are primarily rooted in negative societal attitudes towards them, rather than in disability itself. In the systematic review, Wang et al. 2021 [12] identified factors related to public attitudes towards individuals with disabilities. The authors highlight that knowledge about disability and direct contact with PwD are the most extensively studied factors and are consistently associated with more positive public attitudes towards PwD [12]. Gender has also been shown to play a role in shaping these attitudes, with men demonstrating more negative attitudes towards PwD than women [12]. Moreover, research indicates that attitudes towards persons with intellectual disabilities are less positive than attitudes towards deaf, paralysed, or blind individuals [13].

Babik & Gardner* (*2021) [14] examined factors that influence the development of disability perception in society. The authors proposed a comprehensive model of disability perception that integrates cognitive, affective, and behavioural components of children’s attitudes, emphasising that family, school, and the broader social environment play a critical role in shaping these components. Accordingly, this integrative model conceptualises the perception of disability as a multidimensional process in which a society's cultural context and traditions shape the practices of parents and educational settings [14].

According to Huppert and colleagues (2019), cultural norms influence attitudes towards persons with disabilities [15]. For example, children from more individualistic cultures tend to endorse equitable distributions at an earlier age than those from collectivistic cultures. In contrast, in need-based contexts, children from collectivistic cultures show stronger preferences for equity [15]. These cultural patterns, in turn, shape parental practices and school environments, which play a central role in the formation of children’s attitudes towards PwD. Parental attitudes and practices, such as parenting style, political views, intergroup biases, and attitudes towards disability, combined with the characteristics of the educational environment (e.g. availability of inclusive education, teacher attitudes), jointly determine children’s attitudes towards PwD [14].

### Stigmatising attitudes towards persons with intellectual disabilities

As noted earlier, individuals with intellectual disabilities (ID) are often those towards whom public attitudes are the least positive. They are among the most marginalised and stigmatised groups within the broader population of persons with disabilities, and are frequently targeted by ableist beliefs and practices. According to Jansen-van Vuuren and Aldersey (2020), although some progress has been made, stigma continues to represent a major barrier to the acceptance and inclusion of individuals with intellectual and developmental disabilities across diverse cultural contexts [16]. The authors emphasise that culture plays a critical role not only in shaping the experience of stigma but also in determining effective strategies for promoting acceptance and belonging. Consequently, future anti-stigma interventions should be culturally and contextually responsive, rather than designed as one-size-fits-all solutions [16]. Importantly, ableist attitudes towards individuals with intellectual disabilities are also observed among care providers. Research suggests that stigmatising attitudes held by care providers can negatively affect the quality of support offered to individuals with ID. Furthermore, stigmatisation appears to vary across subgroups of people with ID, as scepticism persists among support staff regarding community inclusion for individuals with high support needs, and care providers often struggle to balance protection with empowerment [9].

### Negative attitudes of care providers towards persons with disabilities

Unfortunately, as noted earlier, negative attitudes towards persons with disabilities are evident even among care providers. Empirical and review evidence indicate that negative or stigmatising attitudes among healthcare professionals are not limited to the general population but may also occur in clinical contexts. A systematic review from 2017 by Pelleboer-Gunnink et al. found that mainstream health professionals’ stigmatising attitudes towards people with intellectual disabilities included limited knowledge, low or overly high expectations, stress, fear, anxiety, differential treatment, and insufficient support for autonomy [9]. Similarly, Bacherini et al. reported in 2021 that physicians’ attitudes towards individuals with intellectual disability may affect access to and quality of healthcare, with unfavourable attitudes particularly evident in affective and behavioural dimensions [10]. Large-scale research on disability attitudes among healthcare providers has also shown that explicit acceptance may coexist with implicit bias against persons with disabilities [17].

While there is a widespread belief that healthcare professionals should demonstrate particularly positive, respectful, and sensitive attitudes towards PwD, and that they generally do so, empirical evidence suggests otherwise. In practice, healthcare professionals often display unfavourable attitudes and misconceptions towards PwD that are comparable to those found in the general population [9].

Negative attitudes towards individuals with disabilities expressed by care providers and health services appear to be among the main factors contributing to reduced accessibility of health services for PwD and to suboptimal quality of treatment. PwD report specific negative attitudes from healthcare providers, including a lack of understanding of disability, limited care and patience (e.g., when healthcare professionals do not explain the disease or the patient’s condition in detail, do not speak slowly, or fail to instruct hearing-impaired individuals appropriately), and disregard, such as addressing a patient in an infantilising manner or communicating solely with a caregiver [18]. This ableist and discriminatory approach, which occurs widely in healthcare services, may result from challenges in care provision, presumably attributed to insufficient education, inadequate knowledge or experience among healthcare professionals, increased workload and work intensity, and facilities that are not disability-friendly [19]. These negative attitudes demonstrated by healthcare professionals can undermine therapeutic relationships, as well as the quality of care and treatment outcomes. In effect, the attitudes of healthcare providers may be considered either facilitators or barriers to PwD accessing and using medical services [9]. Such negative attitudes among healthcare professionals can adversely affect the quality of medical care, particularly in rehabilitation settings [9].

### Measurement of attitudes towards disability

The systematic measurement of attitudes towards disability has a long tradition, with Yuker’s Attitude Toward Disabled Persons Scale (ATDP) representing one of the earliest and most influential instruments [20]. Yuker’s work was important not only because it provided a structured way to assess disability-related attitudes, but also because it contributed to a broader shift from viewing persons with disabilities as passive recipients of care towards recognising autonomy, dignity, and social participation [21, 22]. This historical context is relevant to the present review because contemporary research on healthcare professionals’ attitudes continues to rely on standardised, adapted, or newly developed instruments to capture both explicit evaluations and more subtle forms of exclusion.

Subsequent research expanded the range of tools used to assess attitudes towards persons with disabilities in both general and professional populations [23–25].

Palad et al. identified 31 instruments for measuring disability-related attitudes, most of which demonstrated adequate overall utility, although they varied in disability focus and measurement properties [23]. In healthcare and social care contexts, researchers also frequently use profession-specific or author-developed tools tailored to particular clinical settings and patient populations. This variation in measurement approaches is important for interpreting the comparability and strength of evidence across the studies included in the present review.

### Factors affecting attitudes towards persons with disabilities

As previously mentioned, attitudes towards persons with disabilities are multidimensional and may be shaped by demographic factors (e.g. age, gender, education), social and professional factors, including contact with individuals with disabilities, knowledge, and educational and professional experience [21, 26]. Reviews of the literature consistently identify prior contact with persons with disabilities as the strongest predictor of positive attitudes, particularly when such contact occurs in educational or professional settings [27]. Knowledge level and appropriate professional training also play a significant role. Insufficient training in disability-related issues often leads to uncertainty and increased stress in clinical practice [28, 29].

### Ableism as a source of negative attitudes

In international scholarship, ableism refers to beliefs, practices, and social structures that privilege able-bodied individuals while framing disability as a deficit or deviation from the norm [30, 31]. In healthcare, ableism may be explicit or implicit and can manifest as avoidance, reduced expectations, excessive protectiveness, or communication directed to caregivers rather than to patients [32–35]. In the present review, three recurring patterns of negative attitudes were considered particularly relevant: paternalism, ambivalence, and prejudice.

Paternalism refers to a protective but autonomy-limiting attitude in which persons with disabilities are treated primarily as objects of care rather than as autonomous participants in decision-making. In healthcare contexts, paternalistic attitudes may lead to lowered expectations, excessive control, or insufficient involvement of patients in decisions concerning their own care [9, 30, 31, 36–39].

Ambivalence refers to the coexistence of declared acceptance with discomfort, avoidance, or subtle forms of social distance. It may resemble aversive ableism, in which healthcare professionals consciously endorse inclusion but unconsciously reproduce microforms of exclusion, for example, by avoiding eye contact, directing communication to caregivers, or limiting patient autonomy [40–42].

Prejudice refers to emotionally loaded and stereotype-based reactions towards persons perceived as different. In disability contexts, prejudice may be explicit or implicit and can be reinforced by simplified beliefs about competence, dependence, communication ability, or quality of life [21, 43, 44].

Numerous studies emphasise that a lack of knowledge and experience in working with individuals with intellectual disabilities contributes to the persistence of stigmatising attitudes [9]. Conversely, education based on direct contact with persons with disabilities fosters the development of empathy, understanding, and positive beliefs [12, 45]. According to contact and social learning theories [44], attitudes can be modified through observing positive role models and engaging in authentic interactions.

Accordingly, the literature recommends incorporating disability-related content into medical education programs and creating opportunities for meaningful contact with people with disabilities, as these approaches help reduce negative attitudes and foster more inclusive perspectives [12, 45].

Consistent with the World Health Organization’s International Classification of Functioning, Disability and Health (ICF), disability was understood as a multidimensional and context-dependent phenomenon involving impairments, activity limitations, participation restrictions, and environmental factors that may facilitate or hinder functioning. This understanding is compatible with the UNCRPD approach [3], according to which disability results from the interaction between persons with impairments and attitudinal and environmental barriers that may hinder their full and effective participation in society on an equal basis with others. In this review, physical disability referred to impairments primarily affecting mobility, motor functioning, sensory functioning, or other aspects of physical functioning. Intellectual disability refers, according to international classifications of diseases, to significantly below-average intellectual functioning accompanied by substantial limitations in adaptive functioning or independent living [46, 47]. In this review, “disability in general” was used as an analytical category for studies that examined attitudes towards persons with disabilities broadly, without distinguishing between specific disability types. However, in the final synthesis, the studies meeting the physical disability category concerned sensory disability, specifically deafness or Deaf people. Therefore, this category is referred to as physical/sensory disabilities (PSD) in the Results and Discussion.

Previous systematic reviews have examined attitudes towards selected patient or disability-related groups in different populations and professional contexts. For example, Liu et al. (2012) reviewed health professionals’ attitudes towards older people and older patients, but this literature concerns age-related attitudes rather than disability-specific attitudes [48]. Other reviews have focused on students’ attitudes towards disability in the context of inclusive education rather than healthcare practice (Freer, 2021) [49]. Reviews more directly related to disability have often focused on specific groups or disability categories, such as medical students’ attitudes towards people with intellectual disabilities (Ryan & Scior, 2014) [45], care providers’ perspectives in the field of intellectual disabilities (Pelleboer-Gunnink et al., 2021) [9], or health and social care professionals’ knowledge, attitudes and perceptions towards people with learning disabilities and mental health problems (Ee et al., 2021) [50]. A previous systematic review also examined healthcare students’ and professionals’ attitudes towards patients with physical disability (Satchidanand et al., 2012) [27].

Despite these contributions, the existing evidence remains fragmented by disability type, professional group, and measurement approach. In particular, previous reviews have usually focused on selected disability categories, selected professional or educational groups, or specific clinical contexts. They have not provided a recent comprehensive synthesis focused specifically on healthcare professionals’ attitudes towards persons with disabilities across intellectual disability, physical disability, and disability in general, while also considering factors associated with these attitudes and tools used to measure them.

Therefore, the objective of this study was to examine recent attitudes towards persons with disabilities among healthcare professionals over the past decade (2015–2025), taking into account intellectual disability, physical disability, and disability in general (without specification of type). In addition to the main objective of examining healthcare professionals’ attitudes overall, the study also aimed to:

1. Identify patterns and determinants of attitudes, including factors affecting attitudes.
2. Identify tools for measuring attitudes, including the most recent ones.

## Methods

### Search strategy and selection process

The review of publications included the following four electronic databases: Medline (PubMed), Web of Science Core Collection, ERIC (EBSCO) and ProQuest: Ebook Central, Health & Medical Collection, Publicly Available Content Database. The selection of databases was intended to capture medical, psychological, and social perspectives. Priority was given to databases accessible through institutional subscriptions provided by the first author’s university. The electronic database searches covered English-language publications published from January 2015 to February 2025. Supplementary backward and forward citation searching, including Google Scholar searches, continued until May 2025.

The papers were reviewed independently by the first and second authors, and the data were not obtained from other authors. No review protocol was prospectively registered.

### Data collection process and information sources

In the first stage, the initial database for review was prepared. The first author imported records (*n* = 2033) from four electronic databases (PubMed, Web of Science, ERIC EBSCO, ProQuest) into the Zotero program, in accordance with the predefined search strategy (see search terms), manually removed duplicates, and merged the four databases into a single Excel database (*n* = 1,690).

In the second stage, the review was conducted by two independent researchers (the first and second authors) in accordance with the predefined inclusion and exclusion criteria, and the resulting article lists were discussed and approved by all authors:

- After reviewing the titles of 1,690 articles, 1,397 were excluded. At this point, the second author compiled a database of records independently accepted by both reviewers (n = 293);
- After reviewing the titles and abstracts of 293 articles, the second author created another database of records accepted independently by both authors (n = 131).
- After reviewing 131 full-text reports, the second author created a database of articles accepted for review, from which 15 articles were selected after discussion with the other authors, stating the reasons for excluding the rejected articles.
- In the final stage, the first author reviewed both the reference lists and citations of the 15 accepted articles in the Google Scholar database, selected for its versatility and ease of access to texts, in line with the snowballing approach used by other authors (Freer, 2023). This supplementary backward and forward citation search continued until May 2025. An additional 12 articles were selected and accepted after discussion by all authors, reaching a final number of articles (n = 27).

The overall study identification and selection process was completed in May 2025.

Data from included studies were extracted independently by two reviewers using a standardised Excel form; any discrepancies were resolved through discussion and, when necessary, consultation with the full author team. No attempts were made to contact study authors to obtain additional data.

### Search terms

Search terms were based on the PICOS approach specifying Population, Intervention/exposure, Comparison, Outcome and Study design [51, 52]. PICOS is an acronym used for systematic planning and description of scientific literature search strategies, particularly in systematic reviews and evidence-based medicine (EBM) studies. Each letter stands for a key element of the research question:

- P (Population) – the population or patients affected by the study,
- I (Intervention) – intervention or exposure (e.g. therapy, action, factor),
- C (Comparison) – comparison (if applicable),
- O (Outcomes) – results, effects, study outcomes,
- S (Study design) – types of studies (e.g. clinical, cohort, qualitative studies) [53].

PICOS helps to formulate precise research questions and build effective literature search strategies. The search strategy carried out in four databases (PubMed, ProQuest, Web of Science, and Eric) based on the PICOS model is presented in Appendix 1.

The following search terms were used to search all databases: health personnel OR healthcare professionals OR Staff OR Service-provider AND disabled persons OR persons with disabilities OR intellectual disability OR cognitive dysfunction OR learning disability OR mental disability OR physical disability OR cognitive impairment AND attitude* OR belief* OR bias* OR emotion* OR opinion* OR perception* OR perspective* OR view* OR View* OR prejudice OR social stigma OR stereotyping AND Data collection Method or Survey OR Questionnaire OR interview AND PY 2015–2025.

### Eligibility criteria

The Population, Intervention, Comparison, Outcomes and Study (PICOS) design was used as a framework for formulating eligibility criteria. The comparison component was not applied due to the descriptive nature of the review (i.e., general attitudes of healthcare professionals without a comparison group).

The study population comprised healthcare professionals working within the healthcare system, providing clinical services, and having direct contact with patients. Accordingly, social workers, support staff employed in caregiving services, and administrative or managerial personnel were excluded. Studies involving mixed professional groups were included only if healthcare professionals were analysed as a distinct subgroup.

Our intervention (persons with disabilities or physical disabilities or intellectual disabilities) and the outcome (attitudes towards these groups) encompassed a broad range of attitudinal dimensions, which, in the context of healthcare professionals, could influence the quality of therapeutic relationships. These included positive attitudes based on accurate knowledge about persons with disabilities and their rights, emphasising dignity and values such as respect, empathy, and understanding. In contrast, negative attitudes were characterised by distorted perceptions rooted in prejudice, fear, or stereotypes, which may lead to stigmatisation and lower-quality healthcare delivery.

The review focused on healthcare professionals’ attitudes towards persons with disabilities. For eligibility assessment and data extraction, operational definitions were adopted from MeSH terminology. For final synthesis, studies were classified into three analytical categories: disability in general (GD), physical/sensory disabilities (PSD), and intellectual disability (ID). Studies including participants with multiple disabilities were classified according to the disability category analysed in the original study; when disability types were not analysed separately, the study was classified as disability in general (GD). “Persons with Disabilities” refers to individuals with physical or mental impairments that limit daily living activities and may require special accommodations (term introduced in 1963). The term “Persons with Intellectual Disabilities” refers to individuals diagnosed with significantly below-average intelligence and substantial difficulties in adaptive functioning or independent living (term adopted in 1998, replacing pejorative terms such as “mental deficiency,” “idiocy,” or “mental retardation”). “Mental disabilities” were included only when they co-occurred with or resulted from neurological or physical conditions acquired in adulthood (e.g., stroke or dementia). Studies focusing primarily on psychiatric conditions or mental disorders, such as schizophrenia or depression, were excluded. Although the search strategy and eligibility criteria included physical disability (PD), the studies ultimately included in this category concerned sensory disability, specifically deafness or Deaf people. Therefore, in the final synthesis, this category was labelled physical/sensory disabilities (PSD).

Regarding study design, the review primarily targeted quantitative studies employing standardised measurement instruments. However, mixed-methods studies combining quantitative tools with qualitative approaches, such as interviews or diary methods, were also included. Studies using non-standardised instruments were accepted only if they were clearly derived from validated tools or prior research and contained a clearly defined attitudinal component. Studies employing non-validated attitude measures were included solely when the instruments were structured, outcome-oriented, and reported in peer-reviewed publications. Such studies were rated as having lower methodological quality and were included in narrative synthesis only.

To ensure adequate statistical power and generalisability, only studies with a sample size of at least 50 participants were included. Smaller samples are more susceptible to bias, offer limited external validity, and are often considered ethically problematic in quantitative research [54, 55]. Lower thresholds (around 12–40 participants) are often considered acceptable for pilot or feasibility studies [56, 57], while a minimum of 50 participants has been suggested as the lower bound for reliable statistical analyses in survey-based research [58–60]. Accordingly, pilot and feasibility studies with fewer than 50 participants were excluded, as they do not provide sufficient statistical power or generalisability for systematic synthesis [56, 57].

We limited the review to studies published between January 2015 and May 2025 to capture evidence from the most recent decade covered by the review and to complement earlier reviews that had already synthesised parts of the literature on attitudes towards persons with physical or intellectual disabilities.

All inclusion and exclusion criteria are outlined below.

### Inclusion criteria

1. Population
  Healthcare professionals with direct clinical contact with patients, including physicians, nurses, midwives, paramedics, physiotherapists, and occupational therapists.
  Students or trainees from the above medical professionswho were part of a mixed sample, together with practising healthcare professionals.
2. Attitudinal Outcomes
  Studies assessing healthcare professionals’ attitudes towards persons with disabilities or/and physical disabilities or/and intellectual disabilities.
  Attitudes may be cognitive, affective, or behavioural.
  A focus on general or care-related attitudes, rather than attitudes linked exclusively to specific procedures or interventions.
3. Study Design and Measurement
  Quantitative or mixed-method studies with a quantifiable attitudinal outcome.
  Studies using validated or standardised instruments (e.g., ATDP, SADP) or clearly adapted versions based on such tools.
  Studies using author-developed questionnaires were included only when explicitly grounded in prior peer-reviewed research and when they captured attitudinal components. These studies were classified as lower-quality evidence and included in narrative synthesis only.
  Minimum sample size: ≥50 participants, after exclusions and valid responses, to ensure statistical power and generalisability.
  Peer-reviewed empirical studies published between January 2015 and May 2025.
  Language: Only English-language studies were included due to resource constraints.

### Exclusion criteria

1. Population
  Social care personnel, support staff, administrative workers, or professionals not involved in direct healthcare delivery.
  Studies in which participants were only students or trainees of medical or non-medical professions.
2. Focus
  Studies focused solely on training needs, service delivery logistics, or professional development.
  Attitudes towards colleagues with disabilities.
  Studies addressing attitudes towards individuals with mental illness or psychiatric diagnoses, unless clearly linked to a disability-related condition (e.g., dementia, post-stroke psychosis).
  Studies addressing highly specific topics, such as sexuality, bereavement, or challenging behaviour, when not explicitly framed within general attitudes towards persons with disabilities.
3. Study design
  Purely qualitative studies without quantifiable attitudinal outcomes.
  Pilot or feasibility studies with fewer than 50 participants, due to insufficient statistical power and limited generalisability for a systematic review.
  Tool development or validation studies that do not assess actual attitudes.
  Editorials, reviews, letters, or conference abstracts.
  Non-English-language publications.

### Data items and synthesis method

We extracted data from the included studies covering the following domains: authors, year of publication, and country of study (Reference); aim of the study (Aim of Study); sample characteristics, study design, data collection, and analysis (Sample, Design, Analysis); outcome instruments and effect measures (Tool, Effect Measures); results (Main Findings); STROBE-M score (STROBE-M Score); and risk-of-bias assessment (Risk of Bias). These data were tabulated to enable visual comparison across studies (see Table 1). Required data that were missing or insufficiently reported by the authors were indicated in Table 1 and further examined using the STROBE-M checklist and risk-of-bias assessment. No qualitative analysis software was used. Data extraction, tabulation, and synthesis were conducted manually using structured Excel tables. Given the heterogeneity of study designs, populations, disability categories, measurement tools, and reported outcomes, the findings were collated, summarised, and analysed using narrative synthesis. Determinants of attitudes were first extracted as reported in the included studies and then grouped iteratively during synthesis into broader categories, including contact, knowledge, training, professional experience, demographic factors, professional role, and cultural context. When a study reported multiple determinants, each determinant was coded separately. Disagreements in classification were resolved through discussion among the reviewers.

**Table 1:** Systematic review summary table – healthcare professionals’ attitudes toward persons with disabilities

No	**Reference** **Aim of Study, Sample,** **Design, Analysis**	**Tool, Effect Measures**	**Main Findings**	**STROBE-M score,** **Risk of bias**
1	Alnahdi (2025), Saudi Arabia [61] Aim: Assess healthcare providers’ attitudes toward individuals with ID and identify educational and practice implications. Sample: Healthcare professionals (*N* = 284: doctors, nurses, and other staff). Design: Cross-sectional survey, data collected in person. Analysis: descriptive statistics, ANCOVA.	Tool: Adaptation Attitudes Toward Intellectual Disability ATTID – short form based on the Arabic version. Self-report 35-item on a 5-point Likert scale covering: Discomfort, Knowledge of capacity and rights, Attitudes towards interacting with individuals with ID. Original internal consistency was not reported. Internal consistency in the study sample was good [α_C_ = 0.82–0.86]. Tool not presented in the study. Effect Measures: The ANCOVA models showed acceptable fit to the data for Discomfort (*R*^2^ = 0.49), Knowledge (*R*^2^ = 0.62) and Attitudes (*R*^2^ = 0.57) toward individuals with ID. Models showed significant predictors in areas of Discomfort (job type, education, income, knowing people with ID, having contact with people with ID during sport, recreational activities, family ID), Knowledge (none) and Attitudes (having contact with people with ID during sport, recreational activities) toward individuals with ID.	Nurses reported more discomfort, while doctors with greater exposure showed more inclusive attitudes. Positive personal contact and structured exposure were associated with more favourable views, highlighting the need for experiential training to reduce attitudinal disparities in healthcare. The suggestion that demographic variables are not predictors of attitudes toward people with ID.	Good [75%] Some concerns. Sample size not supported by a priori or post hoc power calculations. The deliberate selection of employees from a single hospital limits the broader relevance of the results. No effect size or CI was reported.
2	Al-Zboon & Hatmal (2016), Jordan [62] Aim: Assess dentists' attitudes toward persons with ID. Sample: Healthcare professionals (*N* = 54: dentists). Design: Cross-sectional survey, data collected in person. Analysis: descriptive statistics, t-test, ANOVA.	Tool: Authors-developed questionnaire. Self-report 21-item on a 5-point Likert scale covering: Learned disposition, Directing feelings, Thoughts, Actions. There was good internal consistency [α_C_ = 0.77–0.86]. The tool was presented in the study. Effect Measures: No differences in attitudes toward people with ID considering gender (*t* = −0.58, *p* = 0.125) or work experience (*F* = 1.42, *p* = 0.264). Total attitude toward people with ID score was considered as high, positive (*M* = 4.80; *SD* = 4.16).	Dentists demonstrated highly positive attitudes toward individuals with intellectual disabilities. No significant differences were found by gender or professional experience. These results highlight a favourable professional stance and suggest a strong foundation for improving inclusive dental services.	Fair [59%] Some concerns. Sample size not supported by a priori or post hoc power calculations. The unknown randomisation method and lack of information about the measurement location limit the broader relevance of the results. No information about interfering factors. No effect size or CI was reported.
3	Breau et al. (2019), Canada [63] Aim: Assess the attitudes of primary care providers (PCPs) towards persons with ID and identify factors that impact these attitudes, like gender, age, professional status and experience with individuals with ID. Sample: Healthcare professionals (*N* = 95: primary care providers, family physicians, family physicians—medicine trainees and advanced practice nursing students). Design: Cross-sectional survey; online method. Analysis: MANOVA for assessing gender, age and experience effects. MANCOVA assessed professional status, controlling for age.	Tool: Community Living Attitudes Scale – Intellectual Disability version (CLAS-ID) Self-report 40-item on a 6-point Likert scale covering: Empowerment, Exclusion, Sheltering, and Similarity. Neither the original internal consistency of the tool nor its internal consistency in the study sample was reported. The tool was not presented in the study Effect Measures: No differences in attitudes toward people with ID covered by gender (*F*_*4*, 90_ = 2.18, *p* = 0.09). Only in scale, empowerment of females, in contrast to males, obtains significantly higher results. Age was considered a significant attitude factor (*F*_94, 16_ = 3.86, *p* < 0,1). Considering age in the model, career stage was not a significant factor to predict attitude level (*F*_4, 88_ = 0.93,* p* = 0.45).	Women show more empathetic and inclusive attitudes than men. Younger providers expressed more inclusive attitudes than older ones. Younger participants were more likely to feel that those with intellectual disabilities are similar to themselves and that individuals with intellectual disabilities should be empowered to take control of their lives. Older participants were more likely to believe that individuals with intellectual disabilities are vulnerable. These findings suggest a generational shift in views. The career stage factor was not significant due to the level of attitudes.	Good to Excellent [82–85%] Some concerns. Sample size not supported by a priori or post hoc power calculations. No measurement validation was presented. No effect size or CI was reported.
4	Campos et al. (2020), Chile [64] Aim: Assess the attitudes of health professionals towards Deafness and its association with sociodemographic and Deaf contact experience. Sample: Healthcare professionals (*N* = 182: dentists, nurses, technicians, physicians, midwives, nutritionists, kinesiologists, psychologists, chemical-pharmaceutical, speech therapist). Design: Cross-sectional survey; data collected in person. Analysis: descriptive stats, t-tests, and Spearman correlations.	Tool: Adapted Attitudes to Deafness Scale (AD) [65]. Self-report 11-item on a 6-point Likert scale covering: Oralism, Awkward interactions, Social acceptance, Vision of disability. Both original internal consistency [α_C_ = 0.71] and internal consistency in the study sample [α_C_ = 0.70] were good. The tool was presented in the study Effect Measures: The global mean showed a neutral attitude toward Deaf people (*M* = 3.92, *SD* = 0.82). Attitudes toward Deaf people correlate negatively with age (*ρ* = –0.24, *p* < 0.01) and academic years (*ρ* = –0.32, *p* < 0.01). No differences in attitude were found by gender, profession, relationship with Deaf people, or sign language training aspects.	Healthcare professionals showed overall neutral attitudes toward Deaf individuals with no significant differences based on gender, profession, training, or contact with Deaf patients. Attitudes were more favourable among younger or less experienced professionals.	Good [78%] Low risk. Sample selected based on the availability of personnel only. Effect size or CI presented selectively.
5	Cashin et al. (2025), Australia [66] Aim: Assessed *ambivalence* and *awareness* related to care for people with Intellectual Disability (ID) and/or Autism Spectrum Disorder (ASD). Sample: Healthcare professionals (*N* = 422: registered nurses). Design: Cross-sectional survey; online snowball sampling method. Analysis: descriptive statistics, t-tests, chi-square tests.	Tool: Caregiving Ambivalence Scale [67] – adapted version for persons with ID/ASD. Self-report 6 items on a 4-point Likert scale. There was good original internal consistency [α_C_ = 0.77–0.91]. Internal consistency in the study sample was not reported. The tool was not presented in the study Effect Measures: Lower ambivalence toward people with ID occurred in the Continuing Professional Development Program Group (CPD) compared to respondents without the program (*t*_420_ = −4.95, *p* < 0.01) 'Every Nurse’s Business CPD Program' group demonstrated the highest level of awareness of reasonable adjustments (χ^2^_1_ = 4.71, *p* = 0.03) and ambivalence	Most nurses lacked undergraduate education, and placements focused on ASD/ID care, highlighting an international training gap. Completion of the Every Nurse’s Business CPD program was significantly associated with greater awareness of reasonable adjustments and lower caregiving ambivalence. Findings support the value of structured CPD in improving inclusive care competencies among nurses.	Good [82–83%] Some concerns. Sample size not supported by a priori or post hoc power calculations. Mixed sample methods. No effect size or CI was reported.
6	Desroches et al. (2019), USA [68] Aim: Identify predictors of nurses' attitudes and emotions toward caring for adults with ID in the United States. Sample: Healthcare professionals (*N* = 248: registered and currently practising nurses). Design: Cross-sectional survey; online method. Analysis: descriptive statistics, t-tests, U-tests, Spearman correlation coefficient, Wilcoxon tests, and regression models.	Tools: 1. Caring for Adults with Disabilities Questionnaire [69] – adapted version. Self-report 30-item on a 6-point Likert scale covering: Attitude toward caring, Positive Emotion, Negative Emotion. Subscale scores are calculated by summing item responses on a visual analogue scale (0–40 per item). The original internal consistency of the tool was not reported. There was good internal consistency in the study sample for the tool [α_C_ = 0.86–0.93]. The tool was not presented in the study 2. Prognostic Beliefs Scale [70]. Self-report 14-item on a 2-point no-yes scale covering beliefs about current/future quality of life for adults with ID. There was good original internal consistency [α_C_ = 0.65–0.85] for the tools. Internal consistency in the study sample was not reported. The tool was not presented in the study Effect Measures: Nurses scored significantly lower for attitudes toward ID vs. physical disability (t = 9.35, p < 0.01). ID-specialist nurses showed more positive attitudes (*t* = 5.47, *p* < 0.01), more positive emotions (t = 4.65, p < 0.01), and fewer negative emotions (*t* = − 5.07, *p* <.01) compared to non-specialist nurses Quality of life beliefs predicted attitude (β = 0.31, *p* < 0.01), positive emotions (β = 0.17, *p* < 0.01), and negative emotions (β = − 0.19, *p* < 0.01). The number of adults with ID cared for predicted negative emotions (β = − 0.15, p = 0.04) Used models explained nurses' attitudes, positive and negative emotions toward adults with ID in 19%, 9% and 8%, respectively.	Nurses generally held attitudes trending from neutral to positive toward caring for adults with ID Nurses held less positive attitudes and emotions toward caring for an adult with ID versus a PD. Nurses who specialise in caring for adults with ID held more positive attitudes and emotions toward caring for adults with ID than non‐intellectual disability nurses. Nurses' beliefs about the quality of life of adults with ID as a predictor of nurses' attitudes and emotions toward caring for adults with ID. The number of adults cared for during the nurse's career was identified as a predictor of negative emotions toward caring for adults with ID.	Good [77–78%] Low risk. The role of the foundation was not explained. Effect size or CI presented selectively.
7	Desroches et al. (2022), Australia [71] Aim: Identify influencing factors of nurses’ attitudes and emotions toward caring for adults with ID. Sample: Healthcare professionals (*N* = 115: nurses). Design: Cross-sectional replication survey; Online snowball sampling method. Analysis: descriptive and inferential analysis, Wilcoxon tests, t-tests or Mann–Whitney U tests. Spearman’s rho and multivariate linear regressions.	Tools: Caring for Adults with Disabilities Questionnaire [69] – adapted version. Self-report 30-item on a 6-point Likert scale covering: Attitude toward caring, Positive Emotion, Negative Emotion. Subscale scores are calculated by summing item responses on a visual analogue scale (0–40 per item). The original internal consistency of the tool was not reported. There was good internal consistency in the study sample for the tool [α_C_ = 0.82–0.89]. The tool was not presented in the study. 2. Prognostic Beliefs Scale [70]. Self-report 14-item on a 2-point no-yes scale covering beliefs about current/future quality of life for adults with ID. The original internal consistency of the tool was not reported. There was good internal consistency in the study sample [α_C_ = 0.89] for the tool. The tool was not presented in the study. Effect Measures: Nurses scored significantly lower for attitudes toward ID vs. physical disability (*t* = −5.47, p < 0.01). No differences between nurses' positive (*t* = −1.60, *p* = 0.11) and negative (*t* = −1.31, *p* = 0.19) emotions toward ID and physical disability. ID nurses had a more positive emotions score compared to mainstream nurses (*U* = 941.50, *p* = 0.03), what cannot be said about the attitude score (*t* = 1.45, *p* = 0.15) and negative emotions (*U* = 1579.00, *p* = 0.12) toward people with ID. Nurses’ beliefs about the quality of life of people with ID (β = 0.36, *p* < 0.01) and the number of adults with ID cared for during career (β = 0.25, *p* < 0.05) were significant predictors explaining 31% of attitudes toward people with ID. The model predicting positive emotion toward people with ID showed frequency of contact with ID people (β = 0.21, *p* < 0.05) and number of adults with ID cared for during career (β = 0.22, *p* < 0.05) as significant, explaining 16% in total. No significant predictors explaining negative nurses' emotions toward people with ID.	Nurses’ attitudes toward caring for adults with ID were significantly less positive compared to adults with PD. Mainstream nurses held less positive emotions toward caring for adults with intellectual disability than intellectual disability nurses. Our findings suggest that worldwide, there is room for improvement in nurses’ attitudes and emotions toward this underserved group. Greater frequency of personal contact with adults with intellectual disability was associated with greater positive attitudes and emotions. In Australia, greater personal contact was predictive of nurses’ positive emotions toward caring for adults with intellectual disability.	Excellent [86%] Low risk. Effect size or CI presented selectively.
8	Desroches & Daudelin (2024), USA [72] Aim: Identify Predictors of Nurses’ Attitudes and Emotions Toward Caring for Adults with Intellectual Disabilities. Sample: Healthcare professionals (*N* = 289: nurses). Design: Cross-sectional survey, online method. Analysis: descriptive and inferential analysis, Pearson’s correlation coefficient.	Tool: Adapted Caring for Adults with Disabilities Questionnaire. Self-report 30-item on a 5-point Likert scale covering: Attitude, Positive emotions and Negative emotions. There was good original internal consistency: Attitude [α_C_ = 0.86], Positive emotions [α_C_ = 0.89] and Negative emotions scale [α_C_ = 0.93]. Internal consistency in the study sample was not reported. The tool was not presented in the study Effect measures: Presented multivariable models explained attitude, positive and negative emotions scores by 27%, 15% and 9%, respectively. Significant predictors of attitude towards people with ID were quality of life beliefs (β = 0.46, p < 0.01) and teamwork (β = −0.29, p < 0.01) Quality of life beliefs (β = 0.27) and age (β = 0.24, p < 0.01) were significant predictors for positive emotions. Also, quality of life beliefs (β = 0.18, p < 0.01), age (β = 0.17, p < 0.01), and ID training (β = 0.16, p < 0.01) were significant in predicting Negative emotions toward people with ID.	Nurses’ quality-of-life beliefs predicted nurses’ attitudes and emotions toward caring for AWID. Higher quality of life scores predicted positive attitudes and emotions and decreased negative emotions. Increasing nurse age predicted greater positive emotions and less frequent negative emotions. Increased training related to intellectual disability (ID) predicted decreased frequency of negative emotions. Increased satisfaction with nursing unit teamwork predicted more positive nurse attitudes toward caring for AWID.	Good to Excellent [82–85%] Low risk. A small response rate limits the generalisation of findings and lowers the power of the analysis. The role of the foundation was not explained.
9	Flynn et al. (2015), United Kingdom [73] Aim: Evaluating communicating caring for. people with cancer and ID Sample: Healthcare professionals (*N* = 83: Oncology or a related field nurses. Design: Cross-sectional survey, online method. Analysis: descriptive statistics, mixed ANOVA.	Tool: Care Perceptions and Attitudes Scale – Author-developed questionnaire. Self-report 12-item on a 5-point Likert scale. Internal consistency in the study sample was not reported. The tool was presented in the study Effect Measures: Statistically significant differences between ID and non-ID conditions across all 12 care perception items (*p* < 0.01), with medium to large effect sizes(*d* = 0.51–1.05). Smaller effect for understanding patients’ circumstances (*d* = 0.46) Previous contact with people with ID and type of vignette was observed in: passed knowledge (*F*_1, 81_ = 7.67, *p* < 0.01), experience (*F*_1, 81_ = 6.99, *p* = 0.01), confidence in meeting with patients with ID (*F*_1, 80_ = 4.31, *p* = 0.04), feeling positive in providing care (*F*_1, 80_ = 11.46, *p* < 0.01), providing appropriate care (*F*_1, 79_ = 6.66, *p* = 0.01) feeling less stressed when providing help (*F*_1, 80_ = 6.25, *p* = 0.01). Interactions occurred also in receiving training (*F*_1, 81_ = 6.38, *p* = 0.01), and understanding the circumstances (*F*_1, 80_ = 4.93, *p* = 0.03.).	Nurses perceived significantly more challenges in caring for cancer patients with intellectual disabilities than those without, including lower confidence in communication, reliance on caregivers, and concern about unmet needs. Prior experience with patients with ID mitigated some of these concerns, highlighting the value of exposure and targeted training.	Good [73–76%] High risk. Casual sampling, not supported by a priori or post hoc power calculations, limits the broader relevance of the results. No measurement used for validation was presented. Selective effect size presentations. Selective data analysis presentation, no post-hoc comparisons or group-specific results.
10	Heydarian et al. (2025), USA [74] Aim: To revise and validate the ATPD scale among health care professionals and examine how health professionals’ attitudes toward people with disabilities. Sample: Healthcare professionals (*N* = 272: clinicians and students of nursing, medicine, clinical psychology, rehabilitation sciences, social work, dentistry, optometry, and other health care professions). Design: Cross-sectional survey, online method. Analysis: descriptive statistics, correlation coefficients, factor analysis.	Tool: Revised Attitudes Toward People with Disabilities (r-ATPD). Self-report 20-item on a 5-point Likert scale. The original internal consistency of the tool was not reported. There was good internal consistency in the study sample [α_C_ = 0.89]. The tool was presented in the study Effect Measures: The three-factor r-ATPD model was presented covering: emotionality and social competence, agency and capabilities and expectations of independence. Intercorrelation between factors was moderate to high (*r* = 0.45–0.67) Social desirability showed to be a positive predictor of r-ATPD scores (β = 0.11–0.17). Quality of interactions with people with disabilities was a negative predictor of r-APTD (β = −0.19 to −0.32) when the quantity of contacts was not correlated. Being a man was associated with higher r-ATPD scores (β = 0.07–0.27). Conscientiousness (β = −0.08 to −0.16), openness (β = 0.01 to −0.18), and agreeableness (β = −0.01–0.18) had negative correlations with r-ATPD scores Authoritarianism (β = 0.11–0.24), social dominance (β = 0.05–0.24) and psychological inflexibility (β = 0.02–0.19) were positively correlated with r-ATPD scores. The last presented model showed a significant r-ATPD predictor: medical model endorsement (β = 0.15–0.30). Regression models explained up to 41% of r-ATPD scores.	Men reported significantly higher disability stigma than women and nonbinary participants. Better-quality contact and higher levels of agreeableness, openness, and conscientiousness were linked to lower stigma. In contrast, psychological inflexibility, authoritarianism, and medical model endorsement predicted more stigmatising attitudes. The validated revised ATPD scale offers strong potential for identifying and addressing stigma-related disparities in healthcare for people with disabilities.	Excellent [92%] Low risk. Convenience sampling could raise concerns about the generalisation of results.
11	Iftikhar et al. (2019), Pakistan [75] Aim: To assess the knowledge and attitude of health care professionals regarding persons with disabilities... Sample: Healthcare professionals (*N* = 85: doctors, nurses and allied health professionals like physiotherapists, psychologists). Design: Cross-sectional survey; data collected in person. Analysis: independent t-test and ANOVA.	Tools: 1. Attitude Towards Disabled Persons Scale – version ATDP-O [20]. Self-report 20-item on a Likert scale. Internal consistency in the study sample was not reported. The tool was not presented in the study 2. Questionnaire regarding knowledge (SAQ-K) – authors-developed questionnaire. The number of items and the response scale were not reported. Internal consistency in the study sample was not reported. The tool was not presented in the study Effect Measures: Attitude toward disabled people didn’t differ by age (*t* = 0.92, *p* = 0.35) and gender (*t* = 0.62, *p* = 0.54) or job-related variables like occupation (*F* = 2.72, *p* = 0.07) and years of experience (*F* = 0.60, *p* = 0.55)	Healthcare professionals in Pakistan demonstrated poor knowledge and generally unfavourable attitudes toward persons with disabilities. Awareness of key concepts, such as the international definition of disability, was low across all professions. While overall attitudes were negative, younger professionals and doctors showed slightly more positive scores than older or allied health staff. Demographic and job-related variables didn’t significantly correlate with attitude and knowledge. The findings highlight the need to integrate disability-focused training into undergraduate medical curricula.	Good [72–74%] Some concerns. Convenient sampling and a lack of information about the measurement location limit the broader relevance of the results. No information about interfering factors. No measurement used for validation was presented. No effect size or CI was reported.
12	Khan et al. (2016), Pakistan [76] Aim: To assess the attitude of medical professionals towards disabled persons. Sample: Healthcare professionals (*N* = 230: doctors and nurses). Design: Cross-sectional survey, data collected in person, purposive sampling. Analysis: descriptive results were reported to explain the questionnaire scores and demographic characteristics.	Tool: Interaction with Disabled Persons (IDP) [77]. Self-report 20-item on a Likert scale. Neither the original internal consistency of the tool nor its internal consistency in the study sample was reported. The tool was presented in the study Effect Measures: Mean attitude score ~ 19.6 (scale range unspecified). Comparisons across age, gender, and profession; younger professionals and doctors showed slightly more favourable attitudes. Exact p-values not reported.	Predominantly negative attitudes were observed among healthcare professionals (mean IDP score ~ 76.7), with 43% scoring in the negative range (> 65). Female participants and doctors showed slightly more negative attitudes than male participants and nurses, respectively. Interestingly, frequent contact with persons with disabilities did not consistently correlate with more positive attitudes. These findings highlight the need for targeted education and attitude-shaping interventions in clinical training programs.	Fair [63–65%] High risk. Sample size not supported by a priori or post hoc power calculations. The unknown sample method lacks information about the measurement location, limiting the broader relevance of the results No information about interfering factors. No measurement used for validation was presented. No effect size or CI was reported (no statistical test used).
13	Kumar et al. (2024), India [78] Aim: To evaluate the knowledge and attitudes of healthcare professionals regarding disabilities in a tertiary care setting. Sample: Healthcare professionals (*N* = 231: nurses, doctors, and support staff, such as attendants and cleaning personnel). Design: Cross-sectional survey; online method. Analysis: chi-square tests, Pearson correlations.	Tool: Authors-developed questionnaire. Self-report 10-item and 8-item scales covering attitudes towards disabled people and knowledge regarding disabled people. There was good internal consistency in the study sample for attitude [α_C_ = 0.81] and knowledge assessment [α_C_ = 0.70] for the tool. The tool was presented in the study. Effect Measures: Significantly more male healthcare providers tend to know about the Vikaas-Day Care scheme for persons with disabled children (*p* < 0.01). Unlikely significantly more women got knowledge about unique disability identification and became familiar with responsibilities for providing accessibility for disabled people in society (*p* < 0.01). In attitudes toward disabled people, only in one domain, genders differ significantly: the best healthcare facility free of cost, where women tend to be more positive about. Occupation differs significantly in disability certification for 21 diseases (knowledge area) where doctors tend to get more knowledge (*p* = 0.02). In attitude domain areas: supply for prosthetic appliance (*p* = 0.01), scholarship education (*p* = 0.03), accessibility for transport (*p* < 0.01), infrastructure (*p* < 0.01), and disabled people having equal social opportunities (*p* < 0.01), nurses tend to be less positive. Knowledge was positively correlated with healthcare providers' attitude toward disabled people (*r* = 0.16, *p* = 0.02).	Healthcare personnel showed overall satisfactory knowledge and attitudes toward persons with disabilities (PwD), with doctors scoring higher in certification knowledge and nurses demonstrating more inclusive attitudes. Male participants had significantly greater awareness of government schemes, while females showed stronger healthcare responsibility understanding and greater support for free care for PwD. Doctors were better informed about disability certification, whereas nurses and support staff more frequently endorsed equal social opportunities These findings highlight the need for targeted legal and policy education to bridge professional and gender-related gaps.	Good [76%] Some concerns. Convenient sampling and a lack of information about inclusion criteria limit the broader relevance of the results. No information about interfering factors. No effect size or CI was reported (no statistical test values).
14	Lucassen et al. (2024), Belgium [79] Aim: To investigate the link between diagnostic labels and social distance on inclusive health representations and practices towards people with ID among healthcare professionals Sample: Healthcare professionals (*N* = 163: general practitioners, medical specialists, nurses) Design: Cross-sectional survey; randomised online method. Analysis: descriptive stats, t-tests, hierarchical regression, mediation/moderation.	Tools: 1. Adapted Intellectual Disability Literacy Scale (IDLS) [80]. Self-report 7-point Likert scale covering: ability to recognise ID symptoms, beliefs about potential causes of ID and beliefs about potential sources of interventions. Neither the original internal consistency of the tool nor its internal consistency in the study sample was reported. The tool was not presented in the study 2. Adopted Questionnaire on Inclusive Health Representations & Practices (QIHRP). Self-report 20-point 5-point Likert scale covering: health care professional representation of people with ID, current professional practices, professional skills and professional responsibility. Original internal consistency was not reported. Internal consistency in the study sample was good [α_C_ = 0.75]. The tool was presented in the study Effect Measures: Inclusive health representation and practices toward people with ID were significantly correlated with social distance (*B* = 1.41 [0.60; 2.23], *p* < 0.01) and label (*B* = 2.59 [0.20, 4.98], *p* = 0.03), controlling for demographic variables like age, gender, profession and gender of people with ID Social distance was also a significant partial mediator (β_indirect_ = 0.75 [0.12, 1.62]) between contact with people with ID and inclusive health representations and practices Labelled or unlabelled vignette wasn’t significantly associated with moderate social distance predicting health representation and practices toward people with ID (β = −0.02 [−1.62, 1.58], *p* = 0.98	The study highlighted the positive link between being in contact with people like Daniel or Sophie (with ID), with cognitive and psychosocial vulnerabilities such as intellectual disability (particularly through employment) and better representations and practices in inclusive health, but also with the reduction of social distance. Thus, raising awareness on how to deal with this type of profile (through direct contact with people with intellectual disabilities) could benefit healthcare professionals, but could also be useful for other types of profiles with cognitive and communication difficulties. The study makes a significant contribution to the literature by filling the knowledge gap on the interaction of associations between diagnostic label, social distance and inclusive representations and practices among healthcare professionals, which is necessary to be able to act on these variables to make healthcare more inclusive.	Good to Excellent [80–85%] Low risk. Sample size not supported by a priori or post hoc power calculations, and lack of information about sample methods limit the broader relevance of the results.
15	Morin et al. (2018), Canada [81] Aim: The 3 objectives: (1) examine health care providers (HCPs) pro-inclusion attitudes toward people with intellectual disabilities (ID), (2) compare these to the general population’s attitudes and (3) examine how HCPs’ attitudes vary as a function of their socio-demographic characteristics. Sample: Healthcare professionals (*N* = 367: physicians, psychiatrists, nurses). Design: Cross-sectional survey; online and paper mixed-methods. Analysis: chi-squared tests, t-tests with bootstrapping used for comparisons. Multiple regression and ANOVA with Bonferroni correction.	Tool: Attitudes Toward Intellectual Disability Questionnaire (ATTID). Self-report 67-item on a 5-point Likert scale assessing 34 vignette-based scenarios that depict individuals with varying levels of intellectual functioning covering: Discomfort, Knowledge of capacity and rights, Interaction, Sensitivity/Tenderness, Knowledge of causes. There was good original internal consistency [α_C_ = 0.92] and test–retest stability [*r* = 0.66–0.90]. Internal consistency in the study sample was not reported. The tool was not presented in the study Effect Measures: Attitudes toward people with ID scores in health care professions were mostly positive in all scales (54.06%—86.55%). Mean scores for healthcare professionals on ATTID subscales were: *Discomfort* = 1.66, *Knowledge of capacity and rights* = 2.08, *Interaction* = 2.61, *Sensitivity* = 2.56, and *Knowledge of causes* = 2.33 Compared to the general population, HCPs scored significantly more negative attitudes toward people with ID in interaction (*t*_1980_ = 10.11, *p* < 0.01) and less negative considering sensitivity (*t*_786.309_ = 16.22, *p* < 0.01) or knowledge of causes (*t*_1980_ = 13.20, *p* < 0.01) Statistically significant predictors besides profession were for interaction: age (*t* = 7.21, β = 0.17, *p* < 0.01), perceived knowledge of ID (*t* = 3.46, β = 0.09, *p* < 0.01), perceived quality of relationship with people with ID (*t* = 9.11, β = 0.22, *p* < 0.01); for sensitivity: age (*t* = 2.88, β = 0.07, *p* < 0.01), perceived knowledge of ID (*t* = 3.21, β = 0.09, *p* < 0.01) and for knowledge of causes: educational attainment (*t* = 3.28, β = 0.11, *p* < 0.01) In the healthcare professional group, variables significantly associated with attitudes on the Discomfort scale included: knowledge about ID (*F*_1, 361_ = 61.28, *p* < 0.01, η^2^ = 0.04), frequency of contact (*F*_1, 367_ = 11.87, *p* < 0.01, η^2^ = 0.03) perceived quality of relationship (*F*_2, 3665_ = 12.99, *p* < 0.01, η^2^ = 0.07) and numbers of patients with ID (*F*_1, 367_ = 6.11, *p* < 0.01, η^2^ = 0.05); for interaction: perceived quality of relationship (*F*_2, 371_ = 10.72, *p* < 0.01, η^2^ = 0.06); for sensitivity: knowledge about ID (*F*_1, 367_ = 14.95, *p* < 0.01, η^2^ = 0.04) and for knowledge of causes: educational attainment (*F*_3, 372_ = 10.40, *p* < 0.01, η^2^ = 0.08) and household income (*F*_2, 339_ = 6.95, *p* < 0.01, η^2^ = 0.04).	At least 54% of healthcare professionals (HCPs) demonstrated attitudes consistent with social inclusion and equal rights toward individuals with intellectual disabilities. Compared to the general population, HCPs showed more positive attitudes on the Knowledge of Causes and Sensitivity/Tenderness dimensions, but less positive attitudes on the Interaction dimension Among HCPs, greater self-rated knowledge and higher quality of contact were associated with more inclusive attitudes. However, lower scores on interaction-related items indicate potential challenges in everyday relational dynamics with individuals with ID.	Good to Excellent [82–88%] Low to none risk. Response rate and mixed data collection method limit the broader relevance of the results.
16	Ong et al. (2017), Australia [82] Aim: Evaluate attitudes, knowledge, skills, and experiences to work with children with intellectual disabilities (ID) to facilitate targeted and effective clinician training towards improvement in patient care. Sample: Healthcare professionals (*N* = 160: nurses, midwives, audiologists, dietitians, occupational therapists, physiotherapists, consultants, professors, registrars, residents). Design: Cross-sectional survey; online method. Analysis: Kruskal–Wallis tests, Mann–Whitney tests with multiple comparison corrections. Correlations coefficients.	Tool: Authors-developed questionnaire. Self-report 48-item mixed quantitative (on Likert scale) and qualitative covering: Professional role of participants, Experiences and attitudes of working with people with ID (quantitative), Knowledge and skills, level of experience, Exposure, needs, preferences in the care, areas of training considered most useful. Neither the original internal consistency of the tool nor its internal consistency in the study sample was reported. The tool was not presented in the study Effect Measures: Negative low and moderate (*r* = −0.25 to −0.47) correlations between years of experience with ratings of experience, knowledge and skill in working with people with ID.	Participants have positive attitudes towards working with individuals with ID and adequate knowledge, skills, and experience to work with children with ID. More experienced clinicians, in all professional groups, were more likely to indicate confidence in handling the special needs of these patients. The small degree of variation in attitudes and experiences, and the largely positive responses towards the needs of patients with ID, most likely reflects the growing recognition of the importance of patient-centred care among all health professions.	Good [70–72%] High risk Lack of control over participant entry into the survey, resulting in a heterogeneous sample, and sample size not supported by a priori or post hoc power calculations, limits the broader relevance of the results. Interfering factors not analysed No measurement used for validation was presented Selective effect size was presented. Selective data analysis was presented.
17	Saleh et al. (2024), Saudi Arabia [19] Aim: To examine the attitudes of healthcare providers in Hai’l City towards people with disabilities. Sample: Healthcare professionals (*N* = 321: physicians, nurses, administrative staff, other professionals). Design: Cross-sectional survey, online method; Analysis: descriptive statistics, t-tests.	Tool: The Scale of Attitudes towards Disabled Persons (SADP) [83]. Self-report 24-item on a 6-point Likert scale covering: Optimism/human rights, Behavioural, Misconceptions, Pessimism/hopelessness. There was good original internal consistency [α_C_ = 0.88] for the tool. Internal consistency in the study sample was not reported. The tool was presented in the study Effect Measures: Overall attitude toward people with disabilities tends to be positive (71,7% of health care providers) Demographic factors like gender (*p* = 0.07), age (*p* = 0.78), education (*p* = 0.56) or work related factors: years of experience (*p* = 0.39), profession (*p* = 0.18), special care for people with disabilities training (*p* = 0.09) or working with people with disabilities (*p* = 0.54) showed no significant relation to attitudes toward people with disabilities.	The results indicated a generally positive attitude of healthcare providers towards people with disabilities. Older providers (over 35 years old) exhibited the highest positivity rate, although differences in age, sex, educational level, years of experience, and professional role were not statistically significant. Conclusion: The study concludes that while healthcare providers generally have positive attitudes towards people with disabilities, there is a critical need for comprehensive training programs to enhance the quality of care provided. Future research should extend to other regions and employ qualitative methods to gain deeper insights into healthcare providers’ attitudes toward and experiences with people with disabilities.	Good [70%] Low risk No effect size or CI was reported, no statistical test values.
18	Santoro (2018), USA [84] Aim: To examine how knowledge and attitudes toward patients with ID may impact analogue clinical decision-making at different stages of physician education and career. Sample: Healthcare professionals (*N* = 77: medical students, physicians, family medicine, internal medicine, paediatrics, gynaecology). Design: Cross-sectional survey; online method Analysis: descriptive statistics, two-factor MANOVA.	Tools: 1. Clinical Vignettes. Randomly distributed with nondisabled, mild ID and moderate ID conditions followed by an author-designed questionnaire on a 6-point Likert scale covering: psychological, social and biological health concerns. Internal consistency in the study sample was not reported. The tool was presented in the study 2. Scale of Attitudes Toward Disabled Persons (SADP) [83]. Self-report 24-item on Likert scale covering: optimism, human rights, behavioural misconceptions, and pessimism/hopelessness. Original internal consistency was good [α_C_ = 0.88–0.91]. Internal consistency in the study sample was not reported. The tool was not presented in the study 3. The authors developed a knowledge questionnaire. Self-reported 16-item multiple-choice scale. The tool was presented in the study Effect Measures: No significant interaction between level of training and patients' level of ID considering knowledge, attitude and clinical decision-making (Pillai’s Trace, *p* = 0.06, *F* = 2.10; Wilk’s λ = 0.84, *p* = 0.05, *F* = 2.14) Additionally, attitudes toward people with ID (*r* = −0.01, *p* = 0.50) and knowledge about ID (*r* = −0.11, *p* = 0.17) did not correlate with the number of areas of the visits Knowledge about ID people correlated with attitude toward people with ID (*r* = 0.20, *p* = 0.04) Among many variables tested, attitude toward ID people correlates significantly with perceived quality of personal interactions with ID individuals (*r* = 0.23, *p* 0.02)	The general outcome of this research found that there was no significant interaction between physicians’ level of training and patients’ level of ID severity when specifically examining those with mild ID, moderate ID, or typical cognitive functioning based on the variables of physician knowledge, attitudes, and analogue clinical decision-making.	Good [76–79%] Some concerns Self-voluntary sample size not supported by a priori or post hoc power calculations limits the broader relevance of the results. No measurement used for validation was presented. No conflict of interest explanation.
19	Şimşek et al. (2020), Turkey [85] Aim: To investigate the attitudes of healthcare professionals in Istanbul towards people with disabilities. Sample: Healthcare professionals (*N* = 704: physicians, nurses, midwives, social workers, psychologists, other professionals). Design: Cross-sectional survey; online voluntary sampling method. Analysis: descriptive statistics, *t*-tests, ANOVA with post-hoc comparisons, and Pearson’s correlations.	Tool: Adapted Attitude Toward Disabled Persons (ATDP) [20]. Self-report 20-item on a 6-point Likert scale. Original internal consistency was not reported. Internal consistency in the study sample was good [α_C_ = 0.73]. The tool was not presented in the study Effect measures: Mean ATDP scale score of the healthcare professionals included in the analysis was determined as 64.95 ± 13.94 (*Mdn* = 65, *Min* = 28, *Max* = 103) Sociodemographic variables like gender (*t* = 2.10, *p* = 0.04), marital status (*t* = −2.14, *p* = 0.03), education (*F* = 10.14, *p* < 0.01), and income (*F* = 6.04, *p* < 0.01) significantly differ in attitude toward disabled people Willingness to take part in training related to the disabled also differs significantly in attitude (*t* = −1.98, *p* = 0.05). Additionally, healthcare providers with different professions scored significantly different attitudes toward the disabled (*F* = 8.97, *p* < 0.01) Working with disabled people (*t* = −0.73, *p* = 0.47), the presence of a disabled person in a close environment (*t* = 0.79, *p* = 0.43) or age (*r* = −0.01, *p* = 0.70) and work experience (*r* = −0.04, *p* = 0.32) have no significant relation with attitude toward the disabled	The mean ATDP score of healthcare professionals indicates generally positive attitudes toward persons with disabilities, with significant variation by gender, marital status, education, income, and professional role. Physicians and allied health professionals (e.g., psychologists, physiotherapists) showed more favourable attitudes than nurses and administrative staff, while willingness to attend disability-related training was also linked to higher scores. No associations were found with age, years of service, or prior personal experience with disability. The authors concluded that although disabled individuals face persistent barriers in accessing healthcare, positive attitudes can be strengthened through integrating disability-focused education into curricula, regular in-service training, and the establishment of dedicated disability units within health institutions.	Good [76–79%] Some concerns. Voluntary and non-probability sample size not supported by a priori or post hoc power calculations limits the broader relevance of the results. No information about interfering factors. No effect size or CI was reported.
20	Strnadova et al. (2019), Czech Republic [86] Aim: To explore medical professionals’ knowledge, attitudes, and experiences related to mothers with ID. Sample: Healthcare professionals (*N* = 175: doctors, obstetricians, paediatricians, nurses, midwives). Design: Cross-sectional survey; online voluntary sampling method. Analysis: descriptive statistics, chi-square test.	Tool: Author-developed questionnaire based on prior literature. Self-report mixed scale. Neither the original internal consistency of the tool nor its internal consistency in the study sample was reported. The tool was not presented in the study. Effect Measures: Medical personnel showed varied attitudes toward mothers with intellectual disabilities (ID), with patterns linked to exposure and professional background Knowledge gaps were clear—53.5% identified the IQ criterion for ID, and just 3–25% described communication approaches adapted to cognitive needs. Attitudes toward motherhood were mixed: 51.2% believed women with ID can “sometimes” be successful mothers, and 28.9% “mostly yes,” with staff in their first 1–5 years more positive (63.6%). However, 30.5% expressed doubts about whether women with ID understand motherhood. Although 89.4% agreed mothers with ID should be able to bring up their children, many believed this required additional support, with 51% specifying third-person help Gender and profession were associated with restrictive views: 54.8% of women versus 41.5% of men supported abortion for pregnant women with ID, and midwives and nurses (68.6%) were more restrictive than obstetricians (51.5%) and paediatricians (42.2%). Most participants (67.1%) also believed women with ID should not independently decide how many children they have.	Respondents expressed mixed views on the parenting capacity of women with ID; some positive, but others questioned parental rights; strong emphasis on the lack of training.	Good [76–79%] High risk. Convenience sampling and a sample size not supported by a priori or post hoc power calculations limit the broader relevance of the results. No measurement used for validation was presented No missing data. explanation. Interfering factors were not considered. No statistical test presentation. No effect size or CI was reported.
21	VanPuymbrouck et al. (2020), USA [17] Aim: To examine providers’ explicit and implicit disability attitudes, interactions between their attitudes, and correlates of explicit and implicit bias. Sample: Healthcare professionals (*N* = 25,006: occupational and physical therapy assistants, diagnosis and treating practitioners, dentists, technologists and technicians, nursing and home health assistants, other health care support). Design: Secondary data used. Analysis: descriptive statistics, paired-samples t-tests, and regression models were used.	Tools: 1. Disability Attitudes – Implicit Association Test (DA-IAT) [87]. Categorisation tasks are congruent and incongruent with stereotypes. Neither the original internal consistency of the tool nor its internal consistency in the study sample was reported. The tool was not presented in the study 2. Author-developed Explicit Disability Attitudes to score perforations rate on a 7-point Likert scale. Neither the original internal consistency of the tool nor its internal consistency in the study sample was reported. Tool not presented in the study Effect Measures: In healthcare providers, group explicit disability attitudes significantly differ from a mean score of 4.00 (t_23.391_ = 70.34, p < 0.01, d = 0.46), just like implicit disability attitudes significantly differ from a mean score of 0.00 (t_22.749_ = 189,84, p < 0.01, d = 1.26). Linear regression models showed to be well fit to data explaining explicit disability attitudes (F_19, 22.555_ = 54.66, p < 0.01, R^2^ = 0.04) and implicit disability attitudes (F_19, 21.799_ = 52.08, p < 0.01, R^2^ = 0.04) In explicit attitudes prediction model: age (β = 0.03, p < 0.05), disability (β = 0.04, p < 0.05), gender (β = −0.07, p < 0.05), race (β = 0.02–0.06, p < 0.05), disable family member (β = −0.04, p < 0.05), disabled friend (β = −0.13, p < 0.05) and political orientation (β = −0.04 to −0.09, p < 0.05) showed to be significant. In implicit attitudes prediction model: age (β = 0.15, p < 0.05), disability (β = 0.04, p < 0.05), gender (β = −0.09, p < 0.05), race (β = −0.02 to −0.06., p < 0.05), disabled family member (β = −0.02, p < 0.05) disabled friend (β = −0.05, p < 0.05) and political orientation (β = −0.06 to −0.12, p < 0.05) occurred as significant.	Although healthcare providers reported low levels of explicit prejudice, their implicit attitudes showed a moderate preference for nondisabled individuals, indicating aversive ableism. Most participants were unaware of their implicit bias. Factors such as age, gender, political views, and personal connections to disability influenced both implicit and explicit attitudes. The study highlights how unconscious bias may affect equitable healthcare delivery for people with disabilities.	Good [76–77%] Some concerns Lack of explanation of primary data sampling and sample size, not supported by a priori or post hoc power, limits the broader relevance of the results. No measurement used for validation was presented. No conflict of interest explanation.
22	Velonaki et al. (2015), Greece [88] Aim: Examine Greek nurses’ knowledge, attitudes and practices toward Deaf people and determine the factors that influence these parameters. Sample: Healthcare professionals (*N* = 200: nurses). Design: Cross-sectional survey, data collected in person. Analysis: descriptive statistics, t-test, ANOVA, Kruskal–Wallis test, Mann–Whitney’s test, chi-square test and correlation coefficients.	Tools: 1. The Attitudes to Deafness Scale (AD) [65]—modified Greek version of the AD. Self-report 27-item on a 5-point Likert scale. Both original internal consistency [α_C_ = 0.77] and internal consistency in the study sample [α_C_ = 0.79] were good The tool was not presented in the study 2. Nurses’ self-efficacy for caring for Deaf – authors' developed questionnaire. Self-report 4-item on a 4-point Likert scale. There was good internal consistency [α_C_ = 0.77]. Tool not presented in the study Effect Measures: Attitudes toward Deaf people were associated significantly with interest in attending education (*t* = 2.31, *p* = 0.02) and Disability (ρ = 0.19, *p* = 0.02). Self-efficacy for caring for the Deaf was associated significantly with avoiding caring for the Deaf (*t* = −6.67, *p* < 0,01), Contact with the Deaf (ρ = 0.36, *p* < 0.01), Emotion on deafness (ρ = 0.17, *p* = 0.03) and feelings from caring for the Deaf (ρ = 0.64, *p* < 0.01).	Interested in attending education scores a higher level of attitude toward the Deaf (more positive). Previous contacts with deaf people and positive feelings from caring increase self-efficacy for caring for the Deaf. Nurses who had never avoided caring for the Deaf scored higher self-efficacy. No significant correlations between attitude, self-efficacy, and demographic variables.	Good [76–77%] Some concerns. Sample size not supported by a priori or post hoc power calculations and lack of information about measurement location limit the broader relevance of the results. No effect size or CI was reported.
23	Weise & Trollor (2018), Australia [89] Aim: To explore attitudes, confidence, and training needs of Australian Public mental health professionals in the area of ID mental health. Sample: Healthcare professionals (*N* = 566: nursing, occupational therapy, psychiatry, psychology, social work, other). Design: Cross-sectional study; online method. Analysis: descriptive statistics.	Tool: Based on the previous tool (Rose et al., 2012), the authors developed a questionnaire—Attitudes towards providing a service to people with an intellectual disability. Self-report scale covering: attitudes toward providing a service to people with ID (10-point Likert scale), confidence level in providing care to people with ID (7-point Likert scale) and training and development (qualitative measure). There was good original internal consistency [α_C_ = 0.82] for the tool. Internal consistency in the study sample was not reported. The tool was presented in the study. Effect Measures: Healthcare providers agreed that people with ID have the right to receive good mental health care as anybody (*Mdn* = 1, *IQR* = 1–1) and that care can improve the quality of their life (*Mdn* = 1, *IQR* = 1–2) Participants reported that part of the mental health care profession is to take care of people with ID (*Mdn* = 1, *IQR* = 1–3). Health care colleagues (*Mdn* = 1, *IQR* = 1–5) and professional bodies (*Mdn* = 1, *IQR* = 1–2) expected mental health care providers to provide care to people with ID Mental health care providers claim that the needs of people with ID can’t be met within mental health care services (*Mdn* = 7, *IQR* = 4.5–9) and that resources in the mental health care system are not able to provide comprehensive services to people with ID (*Mdn* = 4, *IQR* = 3–8).	The study underlines that people with ID should have access to mental health care, as it can be beneficial to them. Results also suggest that the mental health care system in its current condition could not provide a comprehensive service due to a lack of resources. An important key point was that the education of mental care providers in this area should be provided.	Good [70–73%] Some concerns The sample size was not supported by a priori or post hoc power calculations, and the unknown sampling method makes it difficult to generalise. No information about interfering factors.
24	Werner et al. (2017), Israel [90] Aim: To examine primary care physicians’ beliefs and practices for communicating with patients with ID. Sample: Healthcare professionals (*N* = 425: primary care physicians). Design: Cross-sectional survey. Qualitative and quantitative mixed methods. Analysis: descriptive statistics, t-test, and regression.	Tools: 1. Author-developed questionnaire. Utilisation of effective communication principles. Self-report 18-item on a 5-point Likert scale covering: provision of information, preparation for treatments and simplification of information. Moderate internal consistency in the study sample [α_C_ = 0.54–0.69]. The tool was presented in the study 2. Author-developed questionnaire. Attitudes toward the treatment of individuals with ID. Self-report 21-item on 5-point Likert scale covering: negative affect factor, perception of the field of treatment as undesirable, perception of individuals with ID as unable to make decisions and perception of treatment as part of the physician’s role. Internal consistency in the study sample ranged from moderate to good [α_C_ = 0.53–0.78] for the tool. The tool was presented in the study Effect Measures: Attitudes toward treating people with ID correlated with knowledge of ID in domains, negative affect (*r* = −0.16, *p* < 0.01), perception of treatment of ID as undesirable (*r* = −0.21, *p* < 0.01) and perception of treatment of ID as a physician’s job (*r* = 0.20, *p* < 0.01) More positive affect toward people with ID tends to show in groups having 6 or more patients (*t* = 3.43, *p* < 0.05). In this group (caring for 6 or more patients with ID), perception of treatment as a part of the physician’s role was higher (*t* = 2.10, *p* < 0.05). Utilisation of effective communication principles had the strongest correlation with negative effect (*r* = −0.21 – 0.12) and perception of treatment as physicians' role (*r* = 0.16–0.32). The regression model predicting utilisation of effective communication principles covering demographic, job-related variables and attitude toward treatment of people with ID explained up to 16% of communication variation. Age (β = 0.14, *p* < 0.01), number of patients (β = 0.18, *p* < 0.01), negative effect (β = −0.12, *p* < 0.01), perception of treatment as undesirable (β = −0.11, *p* < 0.05) and perception of treatment of people with ID as physician’s job (β = 0.11, *p* < 0.05) predicted significantly provision of information to allow choice Age (β = 0.11, *p* < 0.05), perception of treatment as undesirable (β = −0.19, *p* < 0.05), perception of ID people as unable to make decisions (β = 0.12, *p* < 0.05) and perception of treatment of people with ID as a physician’s job (β = 0.27, *p* < 0.05) predicted significantly the preparation of patients for treatment Gender (β = −0.11, *p* < 0.05), negative effect (β = 0.12, *p* < 0.05) and perception of treatment of people with ID as a physician’s job (β = 0.22, *p* < 0.01) predicted significantly simplified information.	The higher the physicians' subjective knowledge about ID, the more positive their attitude toward the treatment of people with ID. Caring for more patients with ID, physicians had a more positive attitude toward them, and their perception of treating people with ID as part of physicians’ role was higher. The utilisation of effective communication principles correlated with a more positive attitude toward the treatment of people with ID and a higher perception of the treatment of people with ID as part of physicians’ role.	Good [73–75%] Some concerns. The sample size was not supported by a priori or post hoc power calculations, and mixed sample methods limit the broader relevance of the results. Selective effect size presentations.
25	Woodman et al. (2024), Saudi Arabia [91] Aim: To investigate the attitudes of healthcare providers in relation to people with disabilities. Sample: Healthcare professionals (*N* = 1033, occupations were not stated). Design: Cross-sectional survey; online method. Analysis: descriptive statistics, multiple linear regression, and ANOVA.	Tool: Attitudes Toward Disabled Persons (ATDP) [20]. Self-report 20-item on a 6-point Likert scale. Neither the original internal consistency of the tool nor its internal consistency in the study sample was reported. The tool was presented in the study Effect measures: Multiple regression analysis showed that age 24–25 (β = –7.37, p < 0.001) and having ≥ 3 children (β = –9.72, *p* = 0.01) were significantly associated with less positive attitudes toward people with disabilities. The interaction of nationality and number of children was a significant predictor of attitudes toward people with ID (*F*_4, 1013_ = 4.41, *p* < 0.01). Also, Saudi males and females having no children (*F* = 7.53, *p* < 0.01) and having two children (*F* = 5.99, *p* = 0.02) differ significantly in the ATDP score.	Healthcare professionals in Saudi Arabia generally held slightly negative attitudes toward people with disabilities, based on ATDP scores. Younger participants (ages 18–20) and those without children showed significantly more positive attitudes, while those aged 24–25 and with ≥ 3 children scored lower. These findings highlight the need for targeted awareness and educational initiatives aimed at improving disability-related attitudes, especially among specific demographic subgroups of health professionals.	Good [73–74%] Some concerns. Sample size not supported by a priori or post hoc power calculations. The unknown sample method and lack of information about the inclusion/exclusion criteria. No measurement validation was presented.
26	Yi et al. (2020), Republic of Korea [18] Aim: To develop and validate a scale measuring healthcare professionals’ attitudes toward persons with disabilities and explore group differences. Sample: Healthcare professionals (*N* = 993: doctors, nurses, others). Design: Cross-sectional survey; online method. Analysis: exploratory and confirmatory factor analysis (EFA, CFA), t-tests, ANOVA.	Tool: The authors developed the Attitudes towards Persons with Disability in Health Care scale. Self-report 22-item on a 5-point Likert scale covering: behaviours in clinical situations, knowledge and skills, Emotional response, and responsibility of healthcare professionals. There was good internal consistency in the study sample [α_C_ = 0.86–0.89] for the tool. The tool was presented in the study. Effect Measures: Four domain structure was confirmed (χ^2^_(164)_ = 488.76, *p* < 0.01; RMSEA = 0.06; CFI = 0.94). The total reliability of the Attitude towards Persons with Disabilities in Health Care scale was high (α_C_ = 0.89). Education module tends to differ among healthcare professionals in total score (*F* = 10.08, *p* < 0.01), behaviours (*F* = 3.14, *p* = 0.04) and knowledge (*F* = 30.74, *p* < 0.01) domains. Having clinical contact with people with disabilities differed in total attitude score (*t* = −2.17, *p* = 0.03), knowledge (*t* = −6.72, *p* < 0.01) and emotional (*t* = −2.07, *p* = 0.04) domains.	HCPs with disability education scored higher in behaviour and knowledge domains; those with clinical experience scored higher in emotional and knowledge subdomains. Healthcare professionals who participated in an education module showed higher scores in behaviour and knowledge/skills subdomains than those who did not, whereas participants who had clinical experience with persons with disabilities demonstrated higher scores in emotional response and knowledge/skills subdomains.	Good [80%] Some concerns. Sample size not supported by a priori or post hoc power calculations, and lack of information about sampling and measurement location limit the broader relevance of the results. No effect size or CI was reported.
27	Zhao et al. (2022), China [92] Aim: To assess hospital healthcare professionals’ knowledge of dementia and their attitudes toward dementia care Sample: Healthcare professionals (*N* = 603: nurses, doctors) Design: Cross-sectional survey; online and paper mixed-methods. Analysis: t-tests, ANOVA, multiple regression.	Tools: 1. Alzheimer’s Disease Knowledge Scale (ADKS) [93]. Self-report 30-item on true–false scale covering: risk factors, assessment and diagnosis, symptoms, course, life impact, caregiving, treatment and management. Both original internal consistency [α_C_ = 0.73] and internal consistency in the study sample [α_C_ = 0.73] were good. Tool not presented in the study 2. Dementia Attitude Scale (DAS) [94]. Self-report 20-item on a 7-point Likert scale covering: dementia knowledge and social comfort. Both original internal consistency [α_C_ = 0.79] and internal consistency in the study sample [α_C_ = 0.83] were good. The tool was not presented in the study Effect Measures: The overall mean DAS score was 91.3 (SD = 15.9), indicating low levels of positive attitudes toward dementia. In multivariable regression model (*F*_26, 576_ = 4.56, *p* < 0.01, *R*^*2*^_*adj*_ = 0.13) explaining up to 13% of DAS scores training type received (Dementia-related workshops: β = −0.10, *p* = 0.01), length of dementia care (1—3 years: β = −0.08, *p* = 0.05; Over 10 years: β = 0.10, *p* = 0.03), interest in dementia care (β = 0.18, *p* < 0.01) and department (Neurology: β = 0.10, *p* = 0.01; Psychiatric: β = 0.10, *p* = 0.02) were significant predictors of total score The overall mean ADKS score was 20.7 (SD = 2.9), demonstrating limited knowledge of dementia. Besides, one-way analysis of regression models (*F*_21, 581_ = 5.11, *p* < 0.01, *R*^*2*^_*adj*_ = 0.12) tends to explain knowledge and attitudes up to 12.3%. Education (Diploma: β = −0.09, *p* = 0.03; PhD: β = 0.13, *p* < 0.01), experience of searching for dementia-related information (β = 0.16, *p* < 0.01) and willingness to receive dementia education (β = 0.12, *p* = 0.01) were shown to be significant predictors for ADKS scores.	This study found that healthcare professionals in Chinese hospitals demonstrated limited knowledge and only moderately positive attitudes toward dementia. Attitudes varied significantly by profession and department, with geriatric staff and those expressing greater interest in dementia care scoring higher. Regression analyses showed that factors such as training experience, education level, and work exposure significantly predicted both knowledge (ADKS) and attitude (DAS) scores. The findings highlight the need for integrated education and ongoing professional development to improve dementia care competencies among healthcare staff in China.	Excellent [85–86%] Low risk. Selective effect size presentations.

*ASD* Autism Spectrum Disorder, *ID* Intellectual disability, *PwD* Persons with disabilities, *STROBE-M* Strengthening the Reporting of Observational Studies in Epidemiology–Modified

*Statistical abbreviations β* standardised regression coefficient, *CI* Confidence interval, *M* Mean, *Mdn* Median, *N* Sample size, *p* p-value, *r* correlation coefficient, *SD* Standard deviation

The STROBE-M (STROBE-Modified) checklist was used to assess the selected studies, as it offers a structured and quantitative framework for assessing the reporting quality of observational studies (see Table 1). Unlike the original STROBE checklist [95], STROBE-M, developed by Limaye et al. (2018) [96], divides the original 22 items into multiple sub-items, allowing for a more detailed and transparent assessment.

The STROBE-M assessment involved extracting all available information related to reporting quality from each included study, from the title through to end-of-article statements (including funding sources, conflicts of interest, and ethical approval). For each study, all reported outcomes related to healthcare professionals’ attitudes towards persons with disabilities were extracted, regardless of whether they were reported as total scale scores or as specific subscales (e.g., cognitive, affective, or behavioural components). When multiple time points or alternative attitude measures were reported within a single study, the most comprehensive and definitive results were selected for synthesis.

By calculating a percentage score of adherence, STROBE-M allows not only qualitative assessment but also standardised four-point grading of reporting quality from excellent to poor. This ensures comparability across the included observational studies: cross-sectional studies (maximum score = 77 points), a cohort study (84 points), and a case–control study (83 points). STROBE-M supports objective synthesis of evidence in systematic reviews [96]. The STROBE-M score data for selected studies were recorded in Excel format and can be found in Appendix 2.

The STROBE-M checklist was used to assess the completeness and transparency of reporting in the included studies and to inform the risk-of-bias assessment. Each study was independently evaluated by two reviewers working separately: the first and fifth authors assessed studies 1–13, and the first and fourth authors assessed studies 14–27. Item-level assessments were recorded in Excel, and STROBE-M percentage scores were calculated separately for each reviewer. The final assessment was reported either as a single percentage score, when reviewers’ scores were identical, or as a percentage range, when their scores differed.

Furthermore, studies were grouped and synthesised narratively according to:

- the final synthesis category: disability in general (GD), physical/sensory disabilities (PSD), or intellectual disability (ID);
- the type of measurement instrument (validated: Confirmed and tested scales, adapted: Adaptation scales, Author-developed scales and Others) and
- the factors affecting attitudes (Determinants of attitudes: age, gender, contact, knowledge, professional experience, training, profession and specialisation, and other factors).

The choice of narrative synthesis was justified by the heterogeneity of study designs, populations, and measurement instruments, which precluded meta-analytic integration. Potential sources of heterogeneity, such as geographical context, type of professional group, and measurement tools, were explored qualitatively. Sensitivity analyses were not conducted due to the lack of comparable effect size estimates.

Given the descriptive and narrative nature of the review, no formal effect measures (e.g., odds ratios or mean differences) were calculated. Instead, findings were summarised according to the direction of attitudes (positive, neutral, or negative), with particular attention to negative attitudinal patterns (paternalism, ambivalence, and prejudice) and the strength of associations as reported in the original studies.

Publication and reporting bias were minimised using both database searches and manual search strategies, including snowballing. No formal statistical assessment of reporting bias (e.g., funnel plots) was performed due to the non-quantitative (descriptive) nature of the synthesis.

The certainty of evidence for each outcome domain was assessed narratively, considering the methodological quality (STROBE-M score), sample size, and consistency of findings across studies.

## Results

### Study selection

**Fig. 1:**
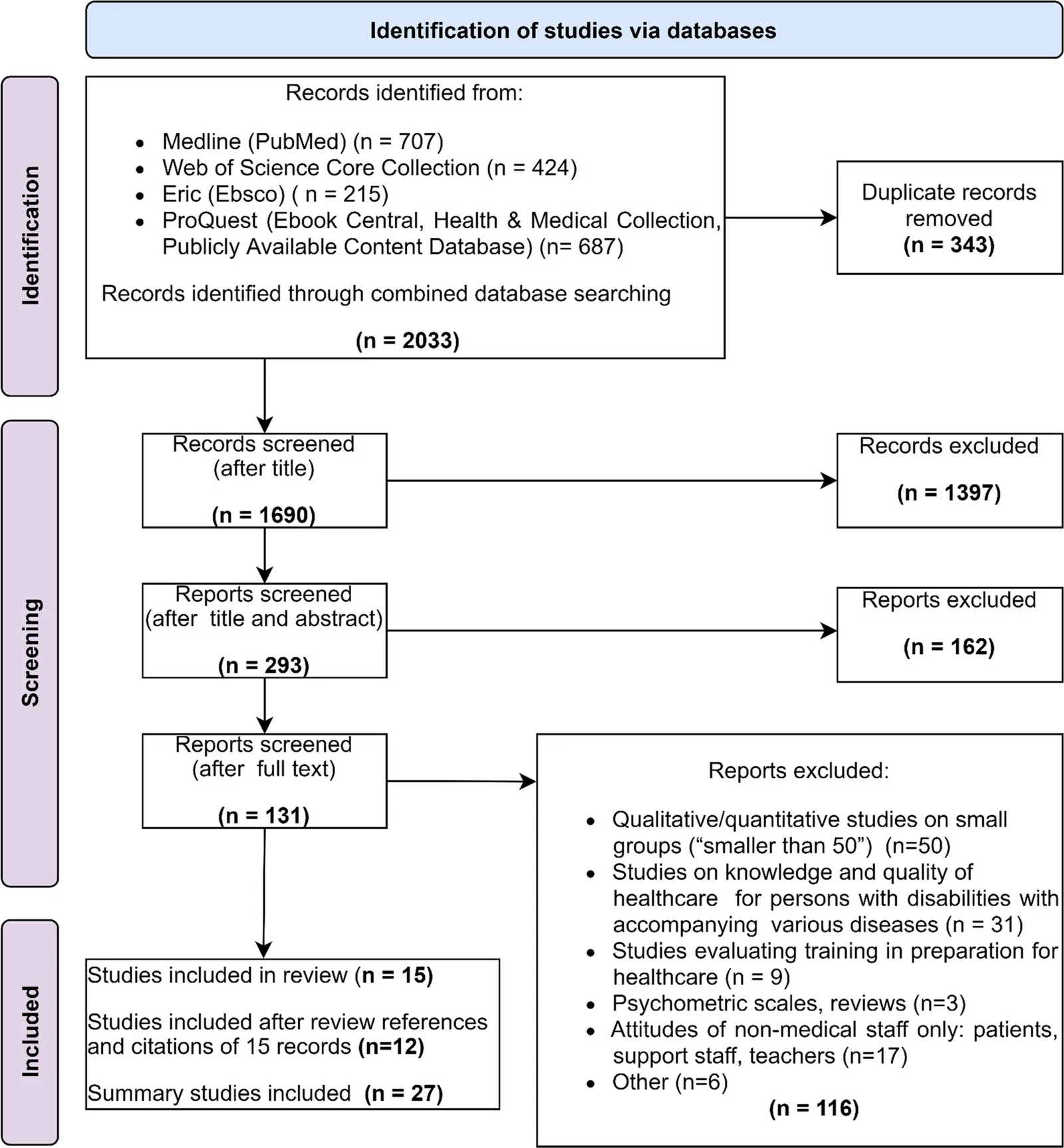
PRISMA flow diagram of the systematic review process for attitudes of healthcare professionals towards persons with disabilities. Source of diagram: https://www.prisma-statement.org/prisma-2020-flow-diagram

The entire review process of the selected literature was performed according to the PRISMA 2020 flow diagram [97] (Fig. 1).

Figure 1 summarises the flow of records through the selection process. During title and abstract screening, records were excluded based on predefined inclusion and exclusion criteria, including, for example, ineligible population, irrelevant outcome, unsuitable study design, non-empirical publication type, language restrictions, and lack of relevance to healthcare professionals’ attitudes towards persons with disabilities. Consistent with the PRISMA 2020 flow diagram, exclusions at this stage are reported as an aggregate number, while detailed exclusion reasons with corresponding numbers are provided in Fig. 1 for the 116 reports excluded after full-text eligibility assessment.

### Study characteristics

Table 1 summarises the key characteristics of the included studies, including study aims, sample characteristics (participants and settings), data collection and analytical methods, measurement instruments, key findings, risk of bias, and STROBE-M assessment.

The quality of the included studies was assessed using the STROBE-M checklist [96], which contains 77 items adapted for observational and cross-sectional research.

Each item was evaluated according to Limaye et al.’s quantitative scoring method: 1 point was assigned for fully satisfied criteria; 0 points for unmet criteria; 2–4 points were used when multiple sub-elements applied; “NA” (Not Applicable) was assigned where a criterion was not relevant to cross-sectional attitudinal studies (e.g., diagnostic criteria, follow-up analyses).

Items marked as “NA” were excluded from the denominator when calculating the final quality percentage, ensuring that only applicable criteria contributed to the total score. The overall quality percentage was computed using the following formula:

$$\begin{aligned} &Quality\;(\%)\\ &=\;(Total\;points\;obtained\;/\\ & \quad Maximum\;possible\;points\;after\;excluding\;NA)\\ & \quad \times\;100 \end{aligned}$$

Study quality was then categorised as follows:

$$\begin{aligned} &\geq85\% - Excellent\;|70-84\%-\;Good\\ &\quad |50-69\%-\;Fair\; | < 50\%-Poor \end{aligned}$$

This approach ensured a proportional and standardised evaluation of methodological quality consistent with the original STROBE-M framework [96]. Among the reviewed studies, seven received an Excellent STROBE-M score. Most of these (*n* = 4) were conducted in North America (Canada and the USA). The remaining top-scoring studies were carried out in Australia (*n* = 1), China (*n* = 1), and Belgium (*n* = 1). The majority of the included studies were rated as Good on the STROBE-M scale. Two studies were evaluated as Fair; both were conducted in the Middle East region – Pakistan (*n* = 1) and Jordan (*n* = 1). Notably, none of the studies was rated as Poor. Across the included studies, the overall methodological quality assessed with the STROBE-M checklist ranged from fair to excellent, with most studies rated as good. The main sources of potential bias were the use of self-reported measures and non-representative sampling strategies.

### Synthesis of attitude patterns across studies

Across the included studies, healthcare professionals’ attitudes were generally neutral to moderately positive, although substantial variation was observed across disability categories, professional groups, and countries. Studies on intellectual disability more frequently reported ambivalence, paternalistic assumptions, lower expectations, uncertainty in communication, or concerns about patients’ autonomy. Studies addressing disability in general showed more mixed findings, including positive explicit attitudes alongside limited knowledge, insufficient preparation, or evidence of implicit bias. The physical/sensory (PSD) disabilities category was represented only by deafness-related studies, limiting the generalisability of findings to physical disabilities more broadly. Across categories, previous contact, higher-quality interactions, disability-related training, greater knowledge, and professional experience were commonly associated with more positive attitudes. Comparability across studies was limited by reliance on self-reported attitudes, non-standardised measures, and heterogeneity in study designs, populations, and outcomes.

Table 1 provides detailed information on all included studies. Each study is described with respect to methodological characteristics (study aim, design, sample, analytical approach, and tools), results (effect measures), and main findings. Table 1 also includes a risk-of-bias assessment and the corresponding STROBE-M score. Most studies employed a cross-sectional design (*n* = 26), with one study based on secondary data analysis. Six studies collected data in person, sixteen collected data online, and two used mixed data-collection methods. The number of observations ranged from *N* = 54 to *N* = 25,006. The main effects, findings, and tools used are described in detail in the Results section of this research.

### Summary of synthesis quality

Given the substantial methodological heterogeneity across study designs, populations, and measurement instruments, the findings were synthesised using a narrative approach. The methodological quality of the included studies ranged from fair to excellent, as assessed using the STROBE-M checklist, with the majority rated as good. The main sources of potential bias were self-reported outcome measures and the use of convenience sampling. Variability in findings was largely attributable to demographic, professional, and cultural differences. No sensitivity analyses or formal statistical assessments of reporting bias were performed. Overall, the certainty of the evidence was judged to be moderate, reflecting the predominance of cross-sectional designs and variability in the psychometric quality of the measurement tools used.

### General overview of included studies

The search strategy yielded 2,033 records, of which 27 studies met the inclusion criteria (see Fig. 1). Table 1 presents detailed characteristics of the included studies.

Studies were conducted across 16 countries representing six global regions (the Middle East, Asia, Europe, North America, South America, and Oceania). The largest number of studies originated from the United States (*n* = 5), followed by Australia (*n* = 4) and Saudi Arabia (*n* = 3). Publication years ranged from 2015 to 2025.

Most of the work involved nursing specialists, including nurses and midwives n = 23 [17–19, 61, 64, 66, 68, 71–76, 79, 81, 82, 85, 86, 88, 89, 91, 92, 98], followed by doctors n = 19 [17–19, 61, 62, 64, 74–76, 79, 81, 82, 84, 85, 85, 86, 89, 90, 92, 99] and dentists [62, 64], general practitioners [99], physicians [64], medicine [74]); and other medical staff and allied health professionals like psychologists, physiotherapists, occupational therapists, dietitians [17–19, 61, 64, 74, 75, 82, 85, 89, 99].

In some studies, the category of healthcare professionals also included nursing students [99] and academic faculty teaching in medical professions [64].

Following the classification, partly used by Palad et al. (2016) [23], studies were grouped into three analytical categories:

1. disability in general (GD), defined as attitudes towards persons with disabilities without specification of disability type (*n* = 9) [17–19, 74–76, 85, 91, 98],
2. physical/sensory disabilities (PSD) (*n* = 2; both studies focused on deafness) [64, 88] and
3. intellectual disability (ID), including mental and developmental forms (n = 16) [61, 62, 66, 68, 71–73, 79, 81, 82, 84, 86, 89, 90, 92, 99].

Throughout the Results and Discussion, GD, PSD, and ID refer to these final synthesis categories. As shown, the majority of studies focused on intellectual disability. The limited number of studies addressing physical or sensory disabilities likely reflects the exclusion of research with purely training-oriented objectives, which have dominated this subfield, thereby limiting generalisability. The scarcity of studies in this area underscores a substantial research gap in understanding healthcare professionals’ attitudes towards physical and sensory disabilities, particularly in comparison with intellectual disabilities. In the final sample, the physical disability category was represented only by studies concerning Deaf participants; therefore, this category was labelled physical/sensory disabilities (PSD), which limits generalisability. Nevertheless, the inclusion of “disability in general” studies provided a broader contextual understanding of healthcare professionals’ attitudes encompassing both physical and intellectual impairments.

### Attitudes towards persons with intellectual disabilities (ID)

Most of the studies included in the review examined healthcare professionals’ attitudes towards persons with intellectual disabilities (ID). Overall, these attitudes ranged from clearly positive and supportive to ambivalent or paternalistic in nature. Several studies also reported subtle forms of prejudice and social distancing, despite declared acceptance and empathy towards individuals with ID. Less positive attitudes towards ID compared to patients with physical disabilities (PD) were reported by Desroches et al. (2019, USA) and Desroches et al. (2022, Australia) [68, 71]. In both studies, nurses expressed generally neutral or moderately positive attitudes towards ID, but these were clearly less positive than their attitudes towards PD. These findings may reflect elements of paternalism, including assumptions about reduced autonomy, as well as subtle cognitive biases related to perceived competence. In another study, Desroches & Daudelin (2024) [72] showed that positive beliefs about the quality of life of people with ID were associated with more empathetic emotional responses, supporting the view that knowledge and positive beliefs can mitigate prejudice.

Qualitative research has revealed a complex picture of clinical attitudes. Flynn et al. (2015) [73] described the experiences of oncology nurses who showed both concern and frustration towards patients with ID, struggling with communication difficulties, patients' dependence on carers, and fears about unmet needs. This is a classic example of emotional ambivalence in caregiving relationships. Similarly, a study by Strnadová et al. (2019) [86] on the parenting skills of women with ID identified the coexistence of supportive and paternalistic beliefs, reflecting doubts about these women’s capacity for independent decision-making.

Despite these challenges, positive attitudes predominate in most studies. Weise & Trollor (2018) [89] demonstrated a high level of acceptance and belief in equal rights to healthcare for individuals with ID, although systemic barriers were identified. Breau et al. (2019) [99] observed positive, inclusive attitudes among doctors and nursing students, highlighting the similarity of persons with ID to the broader population and emphasising empowerment. Women and younger persons presented more progressive views. Santoro 2018 [84] reported moderately positive but heterogeneous attitudes among physicians and medical students, with more favourable views associated with prior contact with individuals with ID, reinforcing the importance of the quality of interpersonal experience.

In a study by Ong et al. (2017) [82], greater clinical experience was associated with a higher sense of competence in working with children with ID. Similarly, Zhao et al. (2022) [92], in their analysis of attitudes towards dementia, identified a comparable pattern—generally positive attitudes coexisting with limited confidence and knowledge, which may reflect a shared mechanism of emotional distancing towards individuals perceived as ‘cognitively dependent’.

Lucassen et al. (2024) [79] reported predominantly positive attitudes of general practitioners, specialists and nurses, with a moderate level of inclusive beliefs. Lower levels of social distance were associated with prior professional experience. In a study by Cashin et al. (2025) [66], participation in the Every Nurse’s Business CPD educational program significantly improved attitudes towards people with ID and ASD (autism spectrum disorder), confirming the effectiveness of education in reducing prejudice.

In Middle Eastern studies [61, 62, 90], physicians tended to express more positive attitudes than other professional groups, possibly reflecting cultural differences in authority structures and social norms. In a study comparing healthcare workers with the general population (Morin et al., 2018) [81], the former group showed higher levels of empathy and concern, but lower willingness to engage in interpersonal interactions, suggesting an ambivalent pattern that combines cognitive acceptance with emotional distance.

In summary, attitudes towards individuals with ID were generally positive but marked by inconsistency. Alongside declarative acceptance, communication difficulties, elements of paternalism, and implicit forms of prejudice persisted, particularly in clinical contexts that require greater patient autonomy, such as oncology, parenting, and dementia.

### Attitudes towards persons with physical/sensory disabilities (PSD)

Only two studies addressed physical/sensory disabilities, both focusing on attitudes towards Deaf individuals. The findings indicated generally neutral to moderately positive attitudes, with no clear evidence of paternalism or overt prejudice. In the study by Campos et al. (2020, Chile) [64], the attitudes of doctors, nurses, midwives and dentists towards deaf persons were neutral and did not vary by gender, profession, or contact with deaf persons. Respondents endorsed the right to healthcare, but highlighted communication-related difficulties. In the study by Velonaki et al. (2015, Greece) [88], no significant relationships were found between attitudes and level of professional experience, education, or self-assessment of competence. Taken together, these studies suggest that while explicit prejudice towards individuals with PSD is uncommon, limited knowledge and lack of contact may create functional barriers in clinical interactions—a subtle form of distance that is not necessarily rooted in negative attitudes.

### Attitudes towards persons with disabilities (disability in general, GD)

Studies examining attitudes towards persons with disabilities without specifying disability type were predominantly conducted in Asian countries and revealed considerable cultural variation. Some studies reported negative attitudes, whereas others identified moderate or clearly positive attitudes, often associated with higher levels of education and prior contact with persons with disabilities.

Negative attitudes were reported in Pakistan [75, 76] and in some studies from Saudi Arabia [91], potentially reflecting entrenched social stereotypes and limited disability-related education. By contrast, positive or moderately positive attitudes were reported in studies from India, Turkey, and Saudi Arabia [19, 85, 98], especially among respondents with higher education and specialist training.

American studies [17, 74] identified the phenomenon of aversive ableism, characterised by the coexistence of declaratively positive attitudes and unconscious bias. The study by VanPuymbrouck et al., involving over 25,000 healthcare workers, showed low levels of explicit prejudice alongside moderate levels of implicit prejudice. Heydarian et al. (2025) [74], using a revised ATDP scale, confirmed moderately positive but heterogeneous attitudes. In both studies, ostensibly accepting declarations coexisted with subtle manifestations of contemporary prejudice, suggesting that even overtly inclusive environments may reproduce hidden forms of ableism, understood as assumptions of able-bodied superiority.

In summary, attitudes towards persons with disabilities were strongly shaped by cultural and cognitive factors. Across many studies, declared acceptance coexisted with implicit prejudices, and, in some cases, with overt paternalistic attitudes towards individuals perceived as “dependent” or “weaker”. These findings underscore the need for sustained anti-discrimination education and ethical reflection within healthcare professions.

### Measures

The reviewed studies used instruments commonly employed to assess attitudes. Self-report questionnaires were the predominant measurement method, reflecting the subjective and multidimensional nature of attitudes towards disability. In several studies, these were supplemented by extended or mixed approaches—such as the use of vignettes or case scenarios representing specific clinical or social situations—to capture respondents’ reactions in a more context-sensitive manner. The measurement tools used across studies are summarised in Table 1.

For this review, they were grouped according to their origin, adaptation process, and psychometric robustness into four main categories:

1. *Confirmed and tested scales;*
2. *Adaptation scales;*
3. *Author-developed scales;*
4. *Other scales.*

#### Confirmed and tested scales

The five standardised scales: SADP, ATTID, STDP, DAS and ADKS were used in several studies, which had a clearly described origin with psychometric values.

Scale of Attitudes Toward Disabled Persons (SADP). Originally proposed by Antonak [83], the scale demonstrates good internal consistency (α_C_ = 0.88–0.91). It was used in two studies [19, 84], although no additional reliability analyses were reported in those studies. The instrument focuses on attitudes towards disability in general and consists of 24 items, conceptualised as a multidimensional measure of attitudes.

Attitudes Toward Intellectual Disability (ATTID). Originally presented by Morin [100], the scale was used in two studies [81] with good internal consistency (α_C_ = 0.82–0.86). The instrument covers multiple dimensions of attitudes towards persons with intellectual disabilities and showed very high reliability in its original validation (α_C_ = 0.92).

The Attitude Toward Disabled Persons Scale (ATDP), provided originally by Yuker [20], was used in three studies [75, 85, 91]. Psychometric values for the original version were not reported, but internal consistency in the study sample (α_C_ = 0.73) was good [85]. Depending on the version, the scale consists of 20–21 items and assesses attitudes towards disability in general.

Dementia Attitude Scale (DAS). Originally presented by O’Connor & McFadden [101], the scale demonstrated good internal consistency (α_C_ = 0.79) and was applied in one study by Zhao [92], where the psychometric value was also confirmed (α_C_ = 0.83). The instrument consists of 20 items and is designed to assess attitudes towards a specific disability domain, namely dementia.

Alzheimer's Disease Knowledge Scale (ADKS). Developed by Carpenter [93]**,** the scale showed good internal consistency (α_C_ = 0.73). It was used in one study [92] with internal consistency confirmed (α_C_ = 0.73). The instrument comprises 30 true–false items and measures multidimensional knowledge related to dementia as a specific disability.

Together, these established instruments provided a solid psychometric foundation, supporting cross-study comparability and confidence in the reliability of the measurements.

#### Adaptation scales

The “Adaptation scales**”** category includes instruments for which clear information on the adaptation process was provided, even when full psychometric properties were not reported. This group comprises six scales, summarised below.

Caring for adults with disabilities questionnaire

An adapted version of this instrument was used in three studies [68, 71, 72] focused on ID. The original version demonstrated good internal consistency (α_C_ = 0.86–0.93), which was also confirmed in the study samples (α_C_ = 0.82–0.93). The tool consists of 30 items and assesses multidimensional attitudes towards disability in general. The Adapted Prognostic Beliefs Scale for adults with ID was originally proposed by Lewis and Stenfert-Kroese [69], showing good internal consistency (α_C_ = 0.65–0.85). It was used in two studies [68, 71]. The psychometric value was also confirmed by internal consistency (α_C_ = 0.89) [71]. The instrument contains 14 items and measures attitudes towards persons with ID.

The Adapted Caregiving Ambivalence Scale for a patient with ID and/or autism was originally proposed by Losada [67], with good original internal consistency (α_C_ = 0.77–0.91). It was used in one study [66]. Psychometric properties were not reported. This is a brief, 6-item instrument designed to capture ambivalent attitudes towards persons with ID.

The Attitudes to Deafness Scale (AD) was proposed by Cooper [65] with good internal consistency (α_C_ = 0.71–0.77) and adapted by [64]. Adaptations also confirm good internal consistency (α_C_ = 0.70). The original 11-item instrument assesses multiple domains related to attitudes towards deafness. Attitudes to deafness scale (AD) was also modified and adapted to the Greek version by Velonaki [88] with good internal consistency (α_C_ = 0.79). This version was based on the original and was expanded to 27 items.

Intellectual Disability Literacy Scale (IDLS), originally presented by Scior & Furnham [80], was adapted and used in one study [79]. The tool was made with 7 items covering the attitude towards persons with ID. No psychometric values were presented.

The Questionnaire on Inclusive Health Representations & Practice (QIHRP), proposed by Rinaldi & Batsele [102] and adapted by Lucassen [79], demonstrated good internal consistency in the study sample (α_C_ = 0.75). The instrument includes 20 items and captures multidimensional aspects of inclusive healthcare practice.

Overall, adapted instruments were typically modified to suit specific cultural or linguistic contexts, while preserving the conceptual structure of the original tools, thereby enabling locally meaningful interpretation without departing from established theoretical frameworks.

#### Author-developed scales

The five author-developed instruments included in this review demonstrated acceptable psychometric quality.

The Revised Attitudes Toward People with Disabilities (r-ATPD) scale was developed by Heydarian [74]**.** Based on ATPD [20], the r-ATPD scale is a shortened 20-item version (selected from the original 30 items). Internal consistency in the study sample (α_C_ = 0.89) confirmed the psychometric value. The scale comprises two main dimensions of the r-ATPD scale and was used to evaluate attitude towards general disability.

The Healthcare Professionals’ Attitudes Towards Persons with Disabilities, developed by Yi [18], demonstrated good internal consistency (α_C_ = 0.86–0.89). The instrument consists of 20 items grouped into four factors and was designed to assess attitudes towards disability in general.

The Attitudes Towards Persons with ID scale, constructed by Al-Zboon & Hatmal [62], showed good internal consistency (α_C_ = 0.77–0.86). This 21-item instrument was designed to assess attitudes towards intellectual disability and was used specifically in research on dentists’ attitudes.

The Attitude and Knowledge Regarding Disabilities scale, developed by Kumar [98], is a brief 8-item instrument designed to assess attitudes towards disability in general. Internal consistency was assessed and found to be good (α_C_ = 0.70–0.81).

The Nurses Self-efficacy for Caring for Deaf scale, developed by Velonaki [88], is a very brief 4-item instrument focusing on a specific area of disability. The scale demonstrated good internal consistency (Cronbach’s α = 0.77).

These instruments were developed to address specific professional or cultural gaps not fully captured by standardised scales. Although several demonstrated acceptable internal consistency, further validation across diverse contexts would enhance their interpretability.

#### Other scales

The final category of measures comprised questionnaires, scales, and procedures for which no detailed information on origin, psychometric values, or other factors was provided.

The Interaction With Disabled Persons scale, proposed by Gathing [77], was used in one study [76]. The instrument consists of 20 items and is intended to assess attitudes towards general disability. No psychometric values were reported. The Attitude Implicit Association Test (DA-IAT), originally proposed by Pruett & Chan [87] was also used in one study [17]. This tool is designed to assess implicit attitudes towards disability in general using categorisation tasks related to stereotypes. No psychometric analyses were reported. The Community Living Attitudes Scale – Intellectual Disability (CLAS-ID), adapted from the original CLAS-MR (Mental Retardation), developed by Henry [103], was used in one study [99]. The scale consists of 40 items covering multiple dimensions of attitudes towards intellectual disability. Psychometric values were not reported. The Attitudes Toward Providing a Service to People with an Intellectual Disability scale, based on the previous version [104], demonstrated good original internal consistency (α_C_ = 0.82) and was used in one study [89]. The instrument combined quantitative and qualitative methods of attitude assessment and focused specifically on attitudes towards persons with ID.

The Utilisation of Effective Communication Principles and Attitudes Toward Treatment of Individuals with ID instruments were developed by Werner [90]. Both scales, Communication Principles and Attitudes, demonstrated moderate to good internal consistency: α_C_ = 0.54–0.69 and α_C_ = 0.53–0.78, respectively. The instruments consist of 18 and 21 items, respectively, and address multiple aspects of working with and caring for individuals with disabilities.

Several of the included studies also used author-developed instruments for which no psychometric properties were reported [73, 75, 82, 86], with mixed quantitative and qualitative methods of attitude assessment [82, 86]. Detailed descriptions of these instruments are provided in Table 1.

Although these “other” instruments offered valuable descriptive insights, their use limits comparability across studies and highlights the need for standardised, validated instruments in future research.

This categorisation enabled the identification of both the most frequently used standardised tools and the areas in which original or locally developed instruments were preferred. The observed variation suggests that while some domains, such as general attitudes towards disability, are commonly assessed using established scales, more specific contexts (e.g., intellectual disability, deafness, or dementia) often require tailored instruments. This pattern points to an ongoing need for context-sensitive measurement tools, alongside greater methodological alignment across studies to enhance the reliability and comparability of findings.

### Determinants of attitudes—factors affecting attitudes

The observed heterogeneity in findings appeared to be primarily related to demographic, professional, and cultural factors.

#### Age

Age was one of the most frequently examined variables differentiating attitudes towards persons with disabilities, although its effects were inconsistent. In relation to intellectual disability (ID), younger general practitioners and medical students in Canada demonstrated more inclusive and autonomy-supportive attitudes than their older counterparts [99]. By contrast, older nurses in the United States reported more positive emotions and fewer negative emotional responses towards persons with ID [72]. In studies addressing disability in general, findings varied across cultural contexts. In Pakistan and Saudi Arabia, younger healthcare workers tended to express slightly more positive attitudes [75, 91], while in other studies the differences were not statistically significant [19, 85]. In the context of PSD, younger healthcare professionals displayed more favourable attitudes towards deaf individuals [64]. Overall, the relationship between age and attitudes was inconsistent across countries and disability types, although younger professionals were more likely to report positive attitudes.

#### Gender

Gender was another factor associated with variation in attitudes. In several studies, women reported more empathetic and inclusive attitudes towards individuals with ID [63, 74], and were also more likely to emphasise the responsibility of the healthcare system to support patients with GD [98]. In studies conducted in the United States, non-binary respondents also expressed more positive attitudes towards disability in general [74]. In some contexts (e.g., Pakistan), however, the opposite pattern was observed, with women displaying less favourable attitudes [76]. In many studies from the Middle East and South America, gender differences were not significant for GD [19, 85] and PSD [64]. Overall, women more frequently reported positive attitudes, particularly in Western countries and in relation to ID, although this pattern was not universal.

#### Contact with persons with disabilities

Personal or professional contact emerged as one of the most consistent predictors of positive attitudes, particularly towards individuals with ID. More frequent and higher-quality contact was associated with greater inclusiveness and reduced social distance towards persons with ID [63, 71, 79, 81]. Similar results were obtained in studies of attitudes towards ID in Australia and Europe [73, 84]. In Arab contexts, positive contact was likewise associated with more supportive attitudes [61]. By contrast, in studies of attitudes towards GD in Pakistan and Turkey, contact did not translate into significant differences [76, 85]. In the case of deafness (PSD), contact was not a significant determinant of attitudes [64, 88]. Overall, the influence of contact appears to be strongest in relation to ID and considerably weaker in relation to deafness.

#### Knowledge

Knowledge and the level of professional preparation consistently differentiated attitudes towards persons with ID. Higher levels of knowledge, regardless of how this knowledge was acquired, were associated with more inclusive attitudes and greater communicative competence [81, 90]. Conversely, insufficient preparation was linked to ambivalence and more negative evaluations in studies examining attitudes towards both GD and ID [75, 86]. In the case of deafness (PSD), the relationship between knowledge and attitudes was not significant [88]. Overall, knowledge appears to play a critical role in shaping attitudes towards intellectual disability, whereas its influence in the context of deafness is less clear.

#### Professional experience

Professional experience, measured by length of practice or frequency of clinical contact with patients with disabilities, was a significant factor differentiating attitudes towards individuals with ID. Longer professional experience was associated with greater clinical confidence and more effective communication skills [79, 82, 90]. In Korea, experience was also linked to improved knowledge and emotional responses to GD [18]. By contrast, this association was less evident in relation to deaf patients (PSD), where professional experience did not appear to differentiate attitudes [64, 88]. In studies from the Middle East, attitudes towards GD [19] also reported no relationship between experience and attitudes. This relationship is therefore mainly evident for ID.

#### Training

Participation in disability-related training emerged as one of the most consistent factors associated with improved attitudes towards persons with ID and, to a lesser extent, towards disability in general (GD). In Australia, a continuing professional development (CPD) program focused on ID and autism spectrum disorders (ASD) increased awareness of patients’ needs and reduced ambivalence [66]. Similar effects were reported in the United States for ID [72], Korea for GD [18] and China for ID [92]. With regard to deafness (PSD), however, training effects were limited unless programs included components on sign language communication [64]. Overall, training appears to be a key determinant of more positive attitudes towards ID, while its impact on sensory disabilities is less consistent.

#### Profession and specialisation

Professional role differentiated attitudes primarily towards persons with ID. General practitioners and other medical doctors more frequently demonstrated inclusive approaches towards ID, particularly when they had greater clinical exposure [61, 63]. By contrast, in Pakistan, studies addressing disability in general did not identify any professional group with consistently more positive attitudes [76]. In the Chilean study focusing on deafness, no significant differences between professional groups were observed [64]. Certain areas of specialisation, such as nursing roles focused on individuals with ID and geriatric wards providing dementia care, were associated with more positive emotional responses and practices [68, 92]. Overall, professional differences were most pronounced in relation to ID and selected clinical specialties.

#### Other factors

Several studies also examined psychological and social determinants of attitudes. Satisfaction with teamwork fostered more positive emotions towards persons with ID [72]. Most factors outside typical demographics were examined in relation to disability in general (GD). Personality traits such as agreeableness and openness were linked to lower levels of stigmatisation, whereas authoritarianism and social dominance orientation were associated with more negative attitudes [74]. In Turkey, higher socioeconomic status was correlated with more positive attitudes [85], while in Saudi Arabia, individuals without children showed higher levels of acceptance [91]. In studies conducted in the United States, political beliefs and levels of implicit bias also played a significant role [17]. In summary, personality and contextual factors—particularly teamwork dynamics, personality traits, and social experiences—substantially shape attitudes towards persons with disabilities, regardless of disability type.

Overall, conclusions regarding generally positive yet ambivalent attitudes and the importance of contact and training are primarily supported by studies rated as good or excellent on the STROBE-M checklist, with findings from fair-quality studies interpreted more cautiously.

## Discussion

### General interpretation of results in the context of existing evidence

This systematic review includes 27 studies examining healthcare professionals’ attitudes towards persons with disabilities, encompassing a range of professional groups and three main disability categories: intellectual disabilities (ID), physical/sensory disabilities (PSD; deafness), and disability in general (GD). The studies were conducted across multiple global regions, primarily North America, Europe, Asia, and the Middle East. The majority of included studies focused on intellectual disability (n = 16). Although the eligibility criteria encompassed both physical and intellectual disabilities, most of the identified research addressed intellectual disability specifically. This pattern likely reflects increasing scholarly attention to the clinical and social complexity associated with intellectual disability, as well as the greater challenges reported by healthcare professionals when working with this group. In contrast, physical and sensory disabilities remain comparatively underrepresented in research on healthcare professionals’ social attitudes.

### Patterns of attitudes depending on the type of disability

The overall pattern of attitudes towards individuals with ID was heterogeneous, ranging from less positive to clearly positive, as demonstrated in multiple studies from the United States, Canada, Western Europe, and Australia [68, 71, 79, 99]. Several studies reported ambivalence and elements of paternalism, particularly in clinically complex contexts, such as assessments of the parenting abilities of women with ID [86] or oncological care [73]. A notable comparative study by Morin et al. found that members of the general population expressed more positive attitudes than healthcare professionals with respect to the behavioural component of attitudes facilitating social integration (the Interaction factor), while healthcare professionals demonstrated greater knowledge and sensitivity. This concerning duality, suggesting reluctance among healthcare staff to engage in direct interaction with patients with ID, has been documented in numerous earlier studies. In a systematic review from 2017, Pelleboer-Gunnink et al. [9] identified stigmatising attitudes towards persons with intellectual disabilities among healthcare professionals, manifested across cognitive dimensions (limited knowledge of ID and resulting unrealistically high or low expectations), affective dimensions (fear, anxiety, and stress), and behavioural dimensions (differential treatment and limited support for the autonomy of persons with ID). Importantly, similar stigmatising attitudes were also observed among professionals specialising in care for individuals with ID, highlighting the scale of the problem [105]

With respect to physical/sensory disabilities (PSD), studies from Chile and Greece [64, 88] reported predominantly neutral attitudes, largely devoid of explicit paternalistic tendencies. This suggests that this category may be perceived as less emotionally charged, with clinical interactions with deaf patients viewed as more technical in nature. Targeted educational modules on communication strategies and Deaf culture were associated with greater acceptance in selected attitudinal domains [64]. Other authors highlight gaps in the education of healthcare professionals in effectively communicating with deaf and hard-of-hearing patients during their professional training [106, 107].

Studies on disability in general have revealed considerable cross-cultural variability. In Pakistan [75, 76] and in some studies from Saudi Arabia [91], negative or ambivalent attitudes prevailed, possibly reflecting local social norms and limited professional exposure to persons with disabilities. In contrast, studies from Turkey [85], Eastern India [98] and the Middle East [19] reported moderately to clearly positive attitudes, particularly among respondents with higher levels of education or formal training. In the United States, the largest review study from 2020 with over 25,000 participants [17] revealed a discrepancy between explicit and implicit attitudes, with openly expressed acceptance coexisting with moderate levels of covert ableism, consistent with the concept of aversive ableism (subtle forms of prejudice). Similar results were obtained by another American researcher five years later as part of work on a revised version of the ATPD scale.

### Paternalism, ambivalence, and prejudice as patterns of negative attitudes

The reviewed studies identified three key patterns shaping healthcare professionals’ attitudes towards persons with disabilities: paternalism, ambivalence, and prejudice, each of which was most evident for different types of disability.

*Paternalism* was most frequently observed in relation to persons with ID—both as beliefs about limited autonomy and competence [73, 86] and as excessive protectiveness disguised as concern. It was less commonly reported in relation to physical/sensory disabilities, specifically deafness, where attitudes were generally neutral rather than overtly patronising [64, 88].

*Ambivalence* was particularly characteristic of attitudes towards ID, with positive declarations coexisting with reservation, uncertainty, or difficulties in communication and relationships (e.g. favourable evaluations of the rights and worth of patients with ID accompanied by difficulties in everyday work [81, 84, 99]. In studies addressing disability in general (GD), ambivalence most often manifests as a gap between declared egalitarian beliefs and underlying ableist assumptions [17, 74]. With respect to deafness (PSD), ambivalence was less pronounced, with findings clustering around neutral attitudes and showing few internal contradictions [64, 88].

*Prejudice* was most evident in studies on GD, especially in South Asian and Middle Eastern countries, where clearly negative attitudes were reported [75, 76, 91]. In Western populations, prejudice more often took the form of contemporary prejudice, characterised by low levels of self-reported explicit prejudice alongside persistent implicit bias [17]. In the context of ID, prejudice was generally less pronounced, but was reflected in greater social distance, lower expectations regarding competence, or a preference for working with other patient groups [68, 79, 81]. The fewest signs of prejudice were observed in two studies focusing on deaf people (PSD), where results were mostly neutral and did not differ significantly across professional groups [64, 88].

### Measures

The diversity of instruments identified in this review reflects broader methodological inconsistencies in research on healthcare professionals’ attitudes towards persons with disabilities. While the frequent use of standardised scales, such as SADP, ATDP, and ATTID, suggests the presence of a shared theoretical foundation, the parallel development and adaptation of author-designed instruments, including tools tailored to specific health conditions, indicate that existing scales do not always capture the full complexity of attitudes within particular professional and cultural contexts. Attitudes are strongly shaped by social and cultural influences, including prevailing norms and values. Furthermore, establishing the cross-cultural validity of these tools may enable comparisons between cultures or population groups, as highlighted in reviews focusing on measurement tools [23]. Similar trends have been noted in other reviews of attitude measurement tools, which emphasise the need to balance cross-study comparability with contextual sensitivity. These authors indicate the importance of developing attitude scales tailored to specific types of disability and/or social groups [24, 35, 45].

### Determinants of attitudes—factors affecting attitudes

An analysis of the included studies shows that contact and professional experience were the most consistent predictors of more positive attitudes, particularly towards individuals with ID [71, 79, 81, 99]. Longer and more intensive clinical exposure, especially in settings that regularly work with individuals with ID, was associated with greater professional confidence, reduced social distance, and more inclusive clinical practices. Knowledge —particularly knowledge related to ID [81, 90]–was linked to more positive attitudes, whereas limited knowledge was associated with ambivalence or negative beliefs; this relationship was less evident for physical/sensory disabilities. In a 2018 review by Appelgren et al. [108] on attitudes towards ID, a lack of awareness and knowledge was noted, which contributed to ID patients being perceived as ‘different’ and reduced the quality of care. Similar findings regarding insufficient staff knowledge were reported in a recent cross-sectional study by Lucassen et al. (2025) [109]. Lack of knowledge about the biomedical causes of intellectual disability (i.e. causes independent of individuals with ID) increased social distance [109]. Conversely, some authors have argued that healthcare professionals’ knowledge of the aetiology of ID is generally higher than that of the general population, even when professionals themselves rate it as insufficient, which may serve as a motivation to develop this knowledge further [81]. Across the reviewed studies, targeted disability-related education—including both undergraduate coursework and continuing professional development programs—consistently led to improvements in attitudes, particularly towards individuals with ID [18, 66, 72, 92].

In summary, the results of this review indicate that healthcare professionals’ attitudes towards individuals with disabilities are generally positive, but are shaped by the type of disability, cultural context, level of professional knowledge and experience, and training. Paternalism and ambivalence were most evident in attitudes towards ID, while prejudice—particularly implicit or culturally conditioned forms—was more prominent in studies addressing disability in general. By contrast, attitudes towards physical/sensory disabilities, represented by deafness, were generally neutral and largely free of strong negative components. Nevertheless, the greatest challenges were associated with intellectual disability, which requires more complex communication skills, heightened sensitivity, and specialised clinical training. The results of numerous reviews [9, 12, 27, 45] indicate that the attitudes of healthcare professionals—particularly towards individuals with ID—are heterogeneous and frequently ambivalent.

Patterns of overprotection and lowered expectations toward persons with disabilities have been described in the literature as forms of discrimination [110]. Additionally, limited confidence in communication and difficulties in working with caregivers have been reported among healthcare professionals working with persons with ID [28]. Unfortunately, current international research continues to document both implicit and explicit forms of ableism among professionals involved in the care of persons with disabilities, as well as limited awareness of ableism itself, which may contribute to discriminatory beliefs and practices [32]. The findings of the present review are consistent with earlier studies, demonstrating that the quality of healthcare professionals’ attitudes is closely linked to professional education, including communication skills, disability-related knowledge, and profession-specific competencies. Deficits in these areas are associated with increased stress, uncertainty, and the persistence of subtle forms of prejudice [27, 105, 108, 111].

Finally, the wide range of instruments used to assess attitudes—from standardised, validated measures to non-validated author-developed tools—while reflecting efforts to adapt measurement to local contexts, limits comparability across studies and hinders cumulative synthesis of findings. These observations underscore the need for a more unified, yet flexible, approach to attitude measurement. The development and validation of tools with demonstrated cross-cultural reliability would facilitate future meta-analyses and international comparisons, thereby supporting more effective educational interventions aimed at reducing prejudice and improving the quality of inclusive healthcare.

### Limitations

The interpretation of the findings of this review requires careful consideration of several methodological and procedural limitations. These limitations concern both the review process itself and the characteristics of the included studies, as outlined below.

#### Limitations of the review process

The review protocol was not registered with PROSPERO or any other registry. Although protocol registration is recommended to increase transparency and reduce the risk of selective reporting, this information was transparently reported in the manuscript and indicated in the PRISMA checklist. Therefore, the lack of registration should be considered a limitation of this review. Despite the use of four major databases, the review was limited to English-language publications, which may have introduced a language bias and resulted in the exclusion of locally published studies in languages other than English. This limitation was partially addressed through the use of snowballing techniques and the inclusion of databases spanning multiple disciplines, including medical, social, and educational fields. Additionally, although two independent reviewers were involved in the study selection process, a risk of subjectivity remains in study eligibility assessment and data extraction. This risk was mitigated through independent evaluations, discussion of discrepancies, and final consensus decisions approved by the team of authors.

#### Limitations of the included evidence

Most of the included studies were cross-sectional, which prevents causal inferences and limits the assessment of the stability of attitudes over time. The findings may therefore reflect temporary opinions rather than enduring attitudes. Furthermore, a substantial proportion of studies relied on self-reported attitude measures, which are vulnerable to social desirability bias—a particularly important issue in research on inclusion and diversity. Only a small number of studies employed instruments designed to assess implicit bias, restricting a more comprehensive understanding of the phenomenon. Another limitation was the considerable diversity of research instruments, ranging from standardised scales to author-developed questionnaires with limited reliability and validity. This methodological diversity hindered comparisons across studies and precluded quantitative analysis (meta-analysis). The uneven representation of disability types also constitutes a limitation: most studies focused on intellectual disability, while research addressing physical disabilities was scarce, which may result in imbalanced generalisations. Although physical and sensory disabilities were underrepresented (with only two studies addressing deafness), this was largely attributable to the exclusion of studies focused on training needs, rather than to a complete absence of research in this area. The category of ‘disability in general’ partially mitigated this gap by capturing broader attitudinal constructs relevant to both physical and intellectual disabilities. Geographical imbalance represents an additional limitation, with most studies originating from North America, South Asia, and Western Europe, and limited representation of low- and middle-income countries, particularly in Eastern Europe and Africa. Finally, several studies failed to report key demographic variables, such as age, educational level, and years of professional experience, thereby limiting a more detailed analysis of potential moderators of attitudes.

### Strengths

The review also demonstrates several strengths that enhance its scientific value. First, it includes a broad range of medical professions, enabling the identification of differences associated with professional roles. In addition, the review considers multiple types of disabilities, which allows for the identification of distinct patterns related to intellectual, physical/sensory, and general disabilities. The review is further distinguished by its comprehensive examination of factors that shape attitudes, including experience, contact, training, age, gender, and educational level, thereby increasing its practical relevance. Furthermore, it addresses the influence of undesirable attitudinal patterns, such as paternalism, ambivalence, and prejudice, including both explicit and implicit forms, allowing for a more multidimensional understanding of the issue. Finally, the inclusion of studies from diverse cultural contexts contributes to a deeper understanding of how systemic and social factors impact healthcare professionals’ attitudes.

### Implications for education, practice, policy, and future research

#### Implications for medical education and clinical practice

The findings suggest that healthcare professionals’ attitudes—particularly towards individuals with intellectual disabilities—are associated with the quality of professional training, prior experience, and perceived competence. These findings support the inclusion of disability-related content in undergraduate medical and nursing curricula, as well as in continuing professional education. Such content may usefully address communication with patients with disabilities, psychosocial support, collaboration with caregivers, and, where relevant, the management of complex clinical interactions. Expanding opportunities for meaningful contact with persons with disabilities—through clinical placements, workshops, and simulation-based learning—may help reduce stereotypes and strengthen professional confidence. It may also be important to strengthen intercultural and anti-ableist competencies, particularly in settings where attitudes have been reported as more negative or ambivalent. The review further suggests that different professional groups may hold distinct attitude profiles, supporting the value of interdisciplinary learning and collaboration within clinical teams. Evidence from recent systematic reviews indicates that well-designed attitude-change interventions can improve attitudes in the short term, although evidence on long-term maintenance and generalisation remains limited [112, 113].

#### Implications for health policy

From a systems perspective, the review findings suggest the need to strengthen disability-related training standards for healthcare staff providing care to persons with disabilities, including both practical and ethical competencies. The findings also highlight the need for policies aimed at reducing systemic barriers to disability-inclusive care, including limited service availability, time constraints in outpatient care, and insufficient organisational support, as highlighted in included studies such as Weise and Trollor (2018) [89]. Attention should also be given to promoting inclusivity and addressing ableism at the institutional level through educational initiatives, structured staff training, and periodic review of policies relevant to disability-inclusive care. Another important implication concerns the need to strengthen and standardise measurement tools, including systematic assessments of reliability and validity. At present, a wide range of scales is in use, and some studies rely on author-developed instruments, which complicates comparisons across countries and professional groups.

#### Implications for future research

The review also highlights several areas that require further research. First, most existing studies are cross-sectional, which limits the ability to assess changes in attitudes over time or following educational interventions; therefore, longitudinal and long-term studies are needed. There is also a clear need to integrate qualitative and mixed-methods approaches, particularly when examining clinical experiences and systemic barriers that may shape attitudes. Studies from the United States have demonstrated substantial discrepancies between explicit and implicit attitudes; conducting similar analyses in other regions could help reveal invisible mechanisms of prejudice, which is why research on implicit ableism is recommended. Greater attention should also be given to physical and sensory disabilities, as the number of studies addressing these areas remains limited, making it difficult to draw broader generalisations. There is additionally a lack of cross-cultural comparisons based on robust psychometric scales, which would make it possible to determine whether differences between countries stem primarily from cultural factors or from methodological variation. Finally, it would be valuable to examine the impact of health policies, educational reforms, and social campaigns on attitudes, as there are currently no studies assessing how systemic changes translate into changes in healthcare professionals’ attitudes.

## Conclusions

The results of this systematic review suggest that healthcare professionals’ attitudes towards persons with disabilities are generally positive but vary considerably across professional groups, world regions, and types of disability. The greatest challenges were observed in relation to intellectual disabilities, where ambivalence, paternalism, and the underestimation of patients’ competencies occurred more frequently.

The factors most consistently associated with more positive attitudes were contact, professional experience, and appropriate training. Cultural variation and differences across professional groups suggest the need for locally tailored educational and policy responses.

Overall, the review suggests that improving the quality and inclusiveness of care for persons with disabilities is likely to require a combination of stronger staff competencies, supportive system-level conditions, and further high-quality research, particularly on implicit bias and the potential impact of educational interventions.

## Supplementary Information

Supplementary Material 1

Supplementary Material 2

Supplementary Material 3

Supplementary Material 4

## Data Availability

The data analysed in this systematic review were obtained from previously published studies. All data generated or analysed during the review are included in the manuscript and supplementary materials (Appendices 1–4).

## Abbreviations

ASD: Autism spectrum disorder
GD: Disability in general (disability not specified by type; final synthesis category)
ID: Intellectual disability
PD: Physical disability (search and eligibility category)
PSD: Physical/sensory disabilities (final synthesis category)
PwD: Persons with disabilities
