# Supplementary material for: Attitudes of healthcare professionals towards persons with disabilities: a systematic review (2015–2025)
Source: BMC Med Educ. 2026 Jul 15;26:1640. doi: 10.1186/s12909-026-09922-0 (PMC13660254; doi:10.1186/s12909-026-09922-0)
Supplement: Supplementary file 1 [file 12909_2026_9922_MOESM1_ESM.docx]

# **Appendix 1. Search Strategies Based on the PICOS Framework**

# *Note. The electronic database searches were completed in February 2025. The database search strings used publication-year limits from 2015 to 2025; therefore, the database searches covered records available in the databases at the time of searching, from January 2015 to February 2025. Supplementary backward and forward citation searching continued until May 2025.*

# **PubMed**

| PICOS Element | Search Terms |
| --- | --- |
| Population | "Health Personnel"[MeSH] OR Staff[TIAB] OR "Service-provider*"[TIAB] OR "Healthcare* professional*"[TIAB] OR "Healthcare* personnel*"[TIAB] OR "Medic* personnel*"[TIAB] |
| Intervention/  Exposure | "Disabled Persons"[MeSH] OR "Intellectual Disability"[MeSH] OR "Cognitive Dysfunction"[MeSH] OR "Disab* person*"[TIAB] OR "Disab* people*"[TIAB] OR "Physical* disab*"[TIAB] OR ("Physical*" AND "challenge*") OR Handicap*[TIAB] OR "Intellectual* disab*"[TIAB] OR "Learning disab*"[TIAB] OR "Mental* disab*"[TIAB] OR "Cognitive disab*"[TIAB] |
| Comparison | Not applicable (general attitudes without comparison group) |
| Outcomes | "Attitude of Health Personnel"[MeSH] OR "Attitude"[MeSH] OR "Knowledge"[MeSH] OR "Health Knowledge, Attitudes, Practice"[MeSH] OR Attitud*[TIAB] OR Belief*[TIAB] OR Bias*[TIAB] OR Emotion*[TIAB] OR Opinion*[TIAB] OR Perception*[TIAB] OR Perspective*[TIAB] OR View*[TIAB] OR "Prejudice"[MeSH] OR "Social Stigma"[MeSH] OR "Stereotyping"[MeSH] |
| Study Design | "Data Collection"[MeSH] OR "Surveys and Questionnaires"[MeSH] OR Questionnaire*[TIAB] OR Interview*[TIAB] OR Survey*[TIAB] OR Psychometric*[TIAB] |
| Timeframe | "2015"[Date - Publication] : "2025"[Date - Publication] |

Note. TIAB refers to the search for text words within the title and abstract;
MeSH refers to the search for Medical Subject Headings, the thesaurus terms that were used in PubMed. **P** (*Population*), **I** (*Intervention*), **C** (*Comparison*), **O** (*Outcomes*), **S** (*Study design*)

# **ProQuest**

| PICOS Element | Search Terms |
| --- | --- |
| Population | "Health Personnel" OR Staff OR ("service provider" OR "service providers") OR "healthcare professionals" OR "Healthcare personnel" OR "Medical personnel" |
| Intervention/  Exposure | "Disabled Persons" OR "Intellectual Disability" OR "Physical disability" OR "Cognitive Dysfunction" OR ("disabled person" OR "disabled persons") OR "Disabled people" OR "Physical* disab*" OR "Physical* challenge*" OR Handicap* OR "Intellectual* disab*" OR ("learning disabilities" OR "learning disability" OR "learning disabled") OR "Mental* disab*" OR ("cognitive disabilities" OR "cognitive disability") |
| Comparison | Not applicable (general attitudes without comparison group) |
| Outcomes | "Attitude of Health Personnel" OR "Attitude" OR "Knowledge" OR "Health Knowledge, Attitudes, Practice" OR Attitud* OR Belief* OR Bias* OR Emotion* OR Opinion* OR Perception* OR Perspective* OR View* OR "Prejudice" OR "Social Stigma" OR "Stereotyping" |
| Study Design | "Data Collection" OR "Surveys and Questionnaires" OR Questionnaire* OR Interview* OR Survey* OR Psychometric* |
| Timeframe | PD(2015-2025) |

**P** (*Population*), **I** (*Intervention*), **C** (*Comparison*), **O** (*Outcomes*), **S** (*Study design*)

# **Web of Science**

| PICOS Element | Search Terms |
| --- | --- |
| Population | TS=("health personnel" OR staff OR "service provider*" OR "healthcare professional*" OR "healthcare personnel" OR "medical personnel") |
| Intervention/  Exposure | TS=("disabled persons" OR "intellectual disability" OR "physical disability" OR "cognitive dysfunction" OR "disabled person*" OR "disabled people" OR "physical* disab*" OR "physical* challenge*" OR handicap* OR "learning disab*" OR "mental disab*" OR "cognitive disab*") |
| Comparison | Not applicable (general attitudes without comparison group) |
| Outcomes | TS=("attitude of health personnel" OR attitude OR knowledge OR "health knowledge" OR "health attitudes" OR "health practice" OR attitud* OR belief* OR bias* OR emotion* OR opinion* OR perception* OR perspective* OR view* OR prejudice OR "social stigma" OR stereotyping) |
| Study Design | TS=("data collection" OR "questionnaires" OR "interviews as topic" OR "survey" OR "questionnaire" OR "interview") |
| Timeframe | PY=(2015-2025) AND Language=English |

**P** (*Population*), **I** (*Intervention*), **C** (*Comparison*), **O** (*Outcomes*), **S** (*Study design*)

# **ERIC**

| PICOS Element | Search Terms |
| --- | --- |
| Population | "health personnel" OR "healthcare professionals" OR "staff" OR "Service-provider" |
| Intervention/Exposure | "disabled persons" OR "persons with disabilities" OR "intellectual disability" OR "cognitive dysfunction" OR "learning disability" OR "mental disability" OR "physical disability" OR "cognitive impairment" |
| Comparison | Not applicable (general attitudes without comparison group) |
| Outcomes | attitud* OR belief* OR bias* OR emotion* OR opinion* OR perception* OR perspective* OR view* |
| Study Design | "Data collection method" OR "survey" OR "questionnaire" OR "interview" |
| Timeframe | PY: 2015–2025; Methodology: Quantitative, Qualitative, Mixed Methods, Survey, Interviews |

**P** (*Population*), **I** (*Intervention*), **C** (*Comparison*), **O** (*Outcomes*), **S** (*Study design*)
