# Supplementary material for: Attitudes of healthcare professionals towards persons with disabilities: a systematic review (2015–2025)
Source: BMC Med Educ. 2026 Jul 15;26:1640. doi: 10.1186/s12909-026-09922-0 (PMC13660254; doi:10.1186/s12909-026-09922-0)
Supplement: Supplementary file 2 [file 12909_2026_9922_MOESM3_ESM.docx]

| **Section and Topic** | **Item #** | **Checklist item** | **Location where item is reported** |
| --- | --- | --- | --- |
| **TITLE** | | |  |
| Title | 1 | Identify the report as a systematic review. | Title page (unnumbered). |
| **ABSTRACT** | | |  |
| Abstract | 2 | See the PRISMA 2020 for Abstracts checklist. | Abstract (page 1); Appendix 4 (PRISMA 2020 for Abstracts checklist). |
| **INTRODUCTION** | | |  |
| Rationale | 3 | Describe the rationale for the review in the context of existing knowledge. | Introduction – Background: Social exclusion as a context for attitudes towards persons with disabilities; Negative attitudes as a factor contributing to marginalisation; Stigmatising attitudes towards persons with intellectual disabilities; Negative attitudes of care providers towards persons with disabilities; Measurement of attitudes towards disability; Factors affecting attitudes towards persons with disabilities; Ableism as a source of negative attitudes (lines 31–213). |
| Objectives | 4 | Provide an explicit statement of the objective(s) or question(s) the review addresses. | Introduction – Background: final paragraph/objectives of the review (lines 214–219). |
| **METHODS** | | |  |
| Eligibility criteria | 5 | Specify the inclusion and exclusion criteria for the review and how studies were grouped for the syntheses. | Methods – Eligibility criteria: Inclusion and Exclusion criteria (lines 274–366). |
| Information sources | 6 | Specify all databases, registers, websites, organisations, reference lists and other sources searched or consulted to identify studies. Specify the date when each source was last searched or consulted. | Methods – Search strategy and selection process; Data collection process and information sources (lines 221–254). |
| Search strategy | 7 | Present the full search strategies for all databases, registers and websites, including any filters and limits used. | Methods – Search strategy and selection process; Data collection process and information sources; Search terms; Appendix 1 (full database search strategies) (lines 221–273 and Appendix 1). |
| Selection process | 8 | Specify the methods used to decide whether a study met the inclusion criteria of the review, including how many reviewers screened each record and each report retrieved, whether they worked independently, and if applicable, details of automation tools used in the process. | Methods – Search strategy and selection process; Data collection process and information sources (lines 221–254). |
| Data collection process | 9 | Specify the methods used to collect data from reports, including how many reviewers collected data from each report, whether they worked independently, any processes for obtaining or confirming data from study investigators, and if applicable, details of automation tools used in the process. | Methods – Data collection process and information sources; Data items and Synthesis Method (lines 231–254 and 367–424). |
| Data items | 10a | List and define all outcomes for which data were sought. Specify whether all results that were compatible with each outcome domain in each study were sought (e.g. for all measures, time points, analyses), and if not, the methods used to decide which results to collect. | Methods – Data items and Synthesis Method; Results – Table 1 (Systematic Review Summary Table – Healthcare Professionals’ Attitudes Toward Persons with Disabilities)  (lines 367–381 and Table 1). |
|  | 10b | List and define all other variables for which data were sought (e.g. participant and intervention characteristics, funding sources). Describe any assumptions made about any missing or unclear information. | Methods – Data items and Synthesis Method; Results – Table 1 (lines 367–381 and Table 1). |
| Study risk of bias assessment | 11 | Specify the methods used to assess risk of bias in the included studies, including details of the tool(s) used, how many reviewers assessed each study and whether they worked independently, and if applicable, details of automation tools used in the process. | Methods – Data items and Synthesis Method: STROBE-M assessment/risk-of-bias assessment; Results – Table 1; Appendix 2 (STROBE-M) (lines 367-424 and Table 1). |
| Effect measures | 12 | Specify for each outcome the effect measure(s) (e.g. risk ratio, mean difference) used in the synthesis or presentation of results. | Methods – Data items and Synthesis Method: effect measures/narrative synthesis; Table 1 (lines 416 - 424 and Table 1). |
| Synthesis methods | 13a | Describe the processes used to decide which studies were eligible for each synthesis (e.g. tabulating the study intervention characteristics and comparing against the planned groups for each synthesis (item #5)). | Methods – Data items and Synthesis Method: eligibility for synthesis and analytical grouping by disability category, measurement instrument, and determinants (lines 382-424). |
|  | 13b | Describe any methods required to prepare the data for presentation or synthesis, such as handling of missing summary statistics, or data conversions. | Methods – Data items and Synthesis Method: data extraction, tabulation, handling of missing/insufficient information; Table 1 (lines 382- 424 and Table 1). |
|  | 13c | Describe any methods used to tabulate or visually display results of individual studies and syntheses. | Methods – Data items and Synthesis Method: tabulation and visual display of included-study characteristics; Table 1 (lines 367- 424 and Table 1). |
|  | 13d | Describe any methods used to synthesize results and provide a rationale for the choice(s). If meta-analysis was performed, describe the model(s), method(s) to identify the presence and extent of statistical heterogeneity, and software package(s) used. | Methods – Data items and Synthesis Method: narrative synthesis and rationale for no meta-analysis (lines 367- 424 and Table 1). |
|  | 13e | Describe any methods used to explore possible causes of heterogeneity among study results (e.g. subgroup analysis, meta-regression). | Methods – Data items and Synthesis Method: qualitative exploration of heterogeneity (geographical context, professional group, measurement tools) (lines 412–415). |
|  | 13f | Describe any sensitivity analyses conducted to assess robustness of the synthesized results. | Methods – Data items and Synthesis Method: sensitivity analyses not conducted due to lack of comparable effect-size estimates (line 415). |
| Reporting bias assessment | 14 | Describe any methods used to assess risk of bias due to missing results in a synthesis (arising from reporting biases). | Methods – Data items and Synthesis Method: publication/reporting bias assessment (lines 399-404, 420–422 and Table 1). |
| Certainty assessment | 15 | Describe any methods used to assess certainty (or confidence) in the body of evidence for an outcome. | Methods – Data items and Synthesis Method: certainty of evidence assessed narratively (lines 423–424). |
| **RESULTS** | | |  |
| Study selection | 16a | Describe the results of the search and selection process, from the number of records identified in the search to the number of studies included in the review, ideally using a flow diagram. | Results – Study selection: PRISMA 2020 flow diagram and description of search/selection process (lines 426–440 and Figure 1). |
|  | 16b | Cite studies that might appear to meet the inclusion criteria, but which were excluded, and explain why they were excluded. | Results – Study selection: reasons for full-text exclusions reported in Figure 1 (lines 426–440 and Figure 1). |
| Study characteristics | 17 | Cite each included study and present its characteristics. | Results – Study characteristics; Table 1 (lines 441–713 and Table 1). |
| Risk of bias in studies | 18 | Present assessments of risk of bias for each included study. | Results – Study characteristics, Synthesis of attitude patterns across studies, Summary of synthesis quality; Table 1: STROBE-M score/risk-of-bias column  (lines 441-500 and Table 1). |
| Results of individual studies | 19 | For all outcomes, present, for each study: (a) summary statistics for each group (where appropriate) and (b) an effect estimate and its precision (e.g. confidence/credible interval), ideally using structured tables or plots. | Results – Study characteristics, Table 1; Study characteristics/main findings/effect measures (lines 441-500 and Table 1). |
| Results of syntheses | 20a | For each synthesis, briefly summarise the characteristics and risk of bias among contributing studies. | Results – Study characteristics; Synthesis of attitude patterns across studies, Summary of synthesis quality; Table 1; Appendix 2 (STROBE-M) (lines 441-500 and Table 1). |
|  | 20b | Present results of all statistical syntheses conducted. If meta-analysis was done, present for each the summary estimate and its precision (e.g. confidence/credible interval) and measures of statistical heterogeneity. If comparing groups, describe the direction of the effect. | Results – Summary of synthesis quality; Synthesis sections by disability category, measures, and determinants (lines 492–814). |
|  | 20c | Present results of all investigations of possible causes of heterogeneity among study results. | Results – Summary of synthesis quality; General overview of included studies; Synthesis sections by disability category, measures, and determinants (lines 492–814). |
|  | 20d | Present results of all sensitivity analyses conducted to assess the robustness of the synthesized results. | Results – Summary of synthesis quality: sensitivity analyses not performed (lines 492–500) |
| Reporting biases | 21 | Present assessments of risk of bias due to missing results (arising from reporting biases) for each synthesis assessed. | Methods – Data items and Synthesis Method: publication/reporting bias assessment; Results – Summary of synthesis quality (lines 492–500). |
| Certainty of evidence | 22 | Present assessments of certainty (or confidence) in the body of evidence for each outcome assessed. | Results – Summary of synthesis quality: certainty of evidence judged narratively/moderate (lines 423, 492–500). |
| **DISCUSSION** | | |  |
| Discussion | 23a | Provide a general interpretation of the results in the context of other evidence. | Discussion – General interpretation of results in the context of existing evidence; Patterns of attitudes depending on the type of disability (lines 816–882). |
|  | 23b | Discuss any limitations of the evidence included in the review. | Discussion – Limitations: Limitations of the included evidence (lines 957–976). |
|  | 23c | Discuss any limitations of the review processes used. | Discussion – Limitations: Limitations of the review process (lines 945–956). |
|  | 23d | Discuss implications of the results for practice, policy, and future research. | Discussion – Implications for education, practice, policy, and future research (lines 988–1030). |
| **OTHER INFORMATION** | | |  |
| Registration and protocol | 24a | Provide registration information for the review, including register name and registration number, or state that the review was not registered. | Limitations, Other information / Supporting information: registration statement; no protocol registered (lines 946, 1044-1049). |
|  | 24b | Indicate where the review protocol can be accessed, or state that a protocol was not prepared. | Methods – Search strategy and selection process; Supporting information: no review protocol was prospectively registered/prepared. (lines 230, 1044). |
|  | 24c | Describe and explain any amendments to information provided at registration or in the protocol. | Not applicable – no registered protocol; therefore, no protocol amendments to report. |
| Support | 25 | Describe sources of financial or non-financial support for the review, and the role of the funders or sponsors in the review. | Other information: Funding statement (line 1073-1074). |
| Competing interests | 26 | Declare any competing interests of review authors. | Other information: Conflicts of interest statement (line 1070-1071). |
| Availability of data, code and other materials | 27 | Report which of the following are publicly available and where they can be found: template data collection forms; data extracted from included studies; data used for all analyses; analytic code; any other materials used in the review. | Other information: Data availability statement / Supporting information (lines 1044–1049). |

*From:* Page MJ, McKenzie JE, Bossuyt PM, Boutron I, Hoffmann TC, Mulrow CD, et al. The PRISMA 2020 statement: an updated guideline for reporting systematic reviews. BMJ 2021;372:n71. doi: 10.1136/bmj.n71. This work is licensed under CC BY 4.0. To view a copy of this license, visit <https://creativecommons.org/licenses/by/4.0/> online: <https://www.prisma-statement.org/prisma-2020>
